# Modeling patient variants of *Cnot1* and *Cdc42bpb* results in distinct forms of congenital diaphragmatic hernia in mice

**DOI:** 10.1093/hmg/ddag106

**Published:** 2026-10-14

**Authors:** Eric L Bogenschutz, Cynthia Carpenter, Ameleen Wong, Kristina Palmer, Aalekh S Mehta, Yannick Ledermann, Caleb Heffner, Kathy J Snow, Carol Bult, Yufeng Shen, Patricia K Donahoe, Samuel P Rowbotham, Frances A High, Wendy K Chung, Stephen A Murray

**Affiliations:** The Jackson Laboratory, 600 Main Street, Bar Harbor, ME 04609, United States; The Jackson Laboratory, 600 Main Street, Bar Harbor, ME 04609, United States; The Jackson Laboratory, 600 Main Street, Bar Harbor, ME 04609, United States; The Jackson Laboratory, 600 Main Street, Bar Harbor, ME 04609, United States; Pediatric Surgical Research Laboratories, Massachusetts General Hospital, 55 Fruit Street Boston, MA 02114, United States; Pediatric Surgical Research Laboratories, Massachusetts General Hospital, 55 Fruit Street Boston, MA 02114, United States; Department of Pediatric Surgery, University Medical Center Mannheim, University of Heidelberg, Theodor-Kutzer-Ufer 1-3, 68167 Mannheim, Germany; The Jackson Laboratory, 600 Main Street, Bar Harbor, ME 04609, United States; The Jackson Laboratory, 600 Main Street, Bar Harbor, ME 04609, United States; The Jackson Laboratory, 600 Main Street, Bar Harbor, ME 04609, United States; Department of Systems Biology, Columbia University Irving Medical Center, 622 West 168th Street New York, NY 10032, United States; Pediatric Surgical Research Laboratories, Massachusetts General Hospital, 55 Fruit Street Boston, MA 02114, United States; Department of Surgery, Harvard Medical School, 25 Shattuck Street Boston, MA 02115, United States; Pediatric Surgical Research Laboratories, Massachusetts General Hospital, 55 Fruit Street Boston, MA 02114, United States; Department of Surgery, Harvard Medical School, 25 Shattuck Street Boston, MA 02115, United States; Pediatric Surgical Research Laboratories, Massachusetts General Hospital, 55 Fruit Street Boston, MA 02114, United States; Department of Surgery, Boston Children’s Hospital, 300 Longwood Avenue Boston, MA 02115, United States; Department of Pediatrics, Massachusetts General Hospital, 55 Fruit Street Boston, MA 02114, United States; Department of Pediatrics, Harvard Medical School, 25 Shattuck Street Boston, MA 02115, United States; Department of Pediatrics, Harvard Medical School, 25 Shattuck Street Boston, MA 02115, United States; Department of Pediatrics, Boston Children’s Hospital, 300 Longwood Avenue Boston, MA 02115, United States; The Jackson Laboratory, 600 Main Street, Bar Harbor, ME 04609, United States

**Keywords:** de novo mutation, disease modeling, genome editing, rare variant association, mouse models, congenital anomalies, congenital diaphragmatic hernia

## Abstract

Congenital diaphragmatic hernia (CDH) is a severe congenital anomaly characterized by impairment of both diaphragm and lung development *in utero*. CDH presents as a spectrum of forms and severities, with diaphragm defects arising in the dorsal/posterior region typically correlating with more severe pulmonary disease and higher risk of mortality than those appearing in ventral/anterior regions. The genetic etiology underlying CDH is complex, with many implicated genes showing variable expressivity and incomplete penetrance in both human patients and mouse models. Here we present *in vivo* validation of two genes previously unassociated with CDH: the CDC42-interacting kinase *CDC42BPB*; and *CNOT1*, a scaffolding protein of the CCR4-NOT protein complex, critical for mRNA regulation through modifications such as deadenylation. Each gene was found to have a damaging, *de novo* missense variant in a recent large-scale CDH patient sequencing screen. Loss of *Cdc42bpb* leads to ventral diaphragmatic hernias, heart septal defects, and minor lung epithelial differentiation defects in mouse embryos. Installation of the orthologous patient-specific missense variant through CRISPR/Cas9 editing leads to less severe ventral diaphragm defects. Mouse embryos with either one or two copies of the orthologous *Cnot1* variant, c.1867C > T (p.R623W), develop dorsal diaphragmatic hernias with low (<50%) penetrance, and mutants showed alterations in mRNA isoform expression consistent with the molecular role of *Cnot1* in RNA splicing. These results underscore the power of *in vivo* functional modeling to validate genes and patient-specific variants uncovered by patient sequencing, reveal two previously unrecognized genetic causes of CDH, and highlight the heterogeneity of different patient anatomic presentations.

## Introduction

Congenital diaphragmatic hernia (CDH) is a relatively common and often deadly congenital anomaly impacting multiple organs of the respiratory system. CDH is characterized by a weakness (eventration) or partial absence of the diaphragm muscle, the latter allowing abdominal contents to enter the thoracic cavity. It is associated with abnormal lung development and function due to both lung-intrinsic and -extrinsic (mechanical) factors [1, 2]. CDH arises in approximately 1 in 2500 live births and results in mortality in an estimated 30–50% of cases [1, 2]. Mortality is due primarily to hypoplastic lung development with incomplete formation of alveoli and respiratory distress at birth [2, 3]. Both human and animal studies have demonstrated that CDH is caused by abnormal development of the embryonic diaphragm and lung, and that *de novo* genetic alterations account for a substantial portion of CDH cases [1, 4, 5].

Exome and genome sequencing efforts have revealed many gene variants associated with human CDH. These variants range in size, inheritance, and recurrence across patients, and include single nucleotide variants (SNVs), small insertions and deletions (indels) and large structural variants (SVs) [1, 6]. However, only approximately 30% of cases have a genetic diagnosis from genomic sequencing [4, 5]. Although a number of causative genes for CDH have been identified and validated through rigorous statistical and/or functional assays, these ongoing discovery efforts continue to identify novel genes and variants, frequently in genes with limited functional annotation. Functional validation to corroborate genotype–phenotype relationships and to gain a better understanding of the biological function of these genes in CDH and normal diaphragm development is crucial for better diagnosis and clinical intervention in CDH.

The diaphragm is a sheet-like skeletal muscle that is unique to mammals and essential for survival due to its role in respiration [7]. The diaphragm develops from two transient embryonic structures: the somites, which are located lateral to the neural tube and give rise to all skeletal muscle of the trunk and limbs, and the pleuroperitoneal folds (PPFs), which are located on the dorsal anterior edge of the developing liver and give rise to the connective tissue of the diaphragm [8]. Muscle progenitors migrate from the somites into the PPFs around mouse embryonic day (E)11.5 [9], as the PPFs start to migrate dorsally and ventrally, spreading across the liver and the mesenchymal septum transversum [9]. As the PPFs migrate, muscle progenitors differentiate into muscle fibers and neuronal projections from the phrenic nerve migrate and branch to form neuromuscular junctions [9, 10]. In mice, the diaphragm is fully formed around E16.5, about 3–4 days before birth [8, 11]. This timing corresponds to approximately E60 in human development, with about 220 days until birth [8, 9]. Genetic studies in mice, through systematic deletion of CDH-causative genes using Cre-drivers targeting critical cell lineages in diaphragm development, suggest that the connective tissue lineage is a key cell population driving CDH development [8, 12–14]. Complementary studies investigating the timing of gene deletion indicate that there are critical windows for when loss of CDH genes correlates with severity of herniation, with early genetic deletion leading to an absence of diaphragm muscle and late deletion leading to normal muscle development [9]. The etiology and likely mechanisms underlying CDH are complex, suggesting many avenues connecting genetic aberrations to overt herniation and potential morbidity.

Similar to other structural congenital anomalies, CDH can present in many forms ranging in clinical severity. CDH can present as the sole structural defect (isolated) or can accompany defects in other organ systems in addition to lung and diaphragm (complex/syndromic). Common comorbidities include congenital heart disease (CHD) and neurodevelopmental disorders, though anomalies can involve multiple other organ systems [2]. The size of the hernia is strongly associated with severity, presumably because a larger diaphragm defect allows more abdominal contents to penetrate the thoracic cavity and mechanically compress the developing lungs. In addition, the location of the diaphragm defect is also important, with dorsal or posterolateral hernias associated with the more severe presentations [1, 3]. Herniations located on the ventral medial edge of the diaphragm are often sub-clinical, presumably due to less impact on lung function, and can go unnoticed by clinicians or patients for many years [1, 3]. The genetics underpinning both size and location of hernias remain understudied, although it has been hypothesized that somatic mosaicism may underlie some forms of CDH in which a herniation occurs in one part of the diaphragm surrounded by correctly patterned muscle [6, 8]. The timing of expression of genes harboring a deleterious variant may also impact the location of the defect, given what is known about diaphragm development. Lung development abnormalities independent of diaphragm defects have also been suggested to drive severity of presentation [2, 15]. This has been termed the ‘dual-hit’ hypothesis, with genetic disruption of lung development serving as the first hit and the mechanical pressure of invading abdominal contents serving as the second hit [15]. This is supported by both genetic and teratogenic models of CDH [2, 15]. Understanding the developmental mechanisms and genetic aberrations that lead to these different forms of CDH remains a critical goal for improving patient diagnosis and outcomes.

To extend our understanding of CDH genetics, we generated mouse models for two novel gene variants associated with CDH identified as part of the Gabriella Miller Kids First Sequencing Initiative [5, 16]. These variants arose within genes (*CDC42BPB* and *CNOT1)* not previously implicated in CDH development, but predicted to be causative for severe, isolated CDH. These genes were prioritized based on known gene function, expression within the developing diaphragm, and previous literature suggesting they are likely to play a critical role in diaphragm development.

*CDC42BPB* encodes the protein MRCKβ (Myotonic Dystrophy Protein Kinase-Like Beta), a serine kinase that acts downstream of the GTPase *CDC42* [17]. CDC42, through MRCKβ and related MRCK family members, is an actin-myosin cytoskeletal regulator, playing a key role in cell motility across multiple cell types [17–19]. CDC42BPB has been shown to directly phosphorylate key cytoskeletal components, such as myosin II regulatory light chain (MLC) [17, 20], and form complexes with other motility-related proteins at leading edges of cell migration [21]. In humans, *CDC42BPB* haploinsufficiency has been associated previously with neurodevelopmental defects, specifically Chilton-Okur-Chung syndrome, an autosomal dominant condition characterized by structural brain and neurodevelopmental disorders [22].

*CNOT1* encodes a critical scaffold protein for the RNA regulation complex CCR4-NOT (Carbon Catabolite Repression 4-Negative On TATA-less). The CCR4-NOT complex, including CNOT1, is highly evolutionarily conserved [23]. The protein complex has been associated with many critical functions of mRNA regulation and turnover, including transcript initiation, adenylation of mRNA, and ubiquitylation [24]. Through serving as scaffolding to other enzymatic CNOT protein family members, CNOT1 has been shown to be essential for the deadenylation function of the complex and overall cell viability [25]. Due to this critical role, loss of CNOT1 in the mouse is embryonic lethal [26], though tissue-specific deletion of CNOT1 has been used to study its function in liver [27] and heart [26]. *De novo* mutations in *CNOT1* have been associated previously with neurological developmental disorders [28–30], including holoprosencephaly, and pancreatic agenesis [31]. Single nucleotide polymorphisms (SNPs) in the *CNOT1* locus have also been associated with cardiovascular disease [32, 33] and pediatric leukemia [34] via GWAS studies. These collective findings demonstrate the pleiotropic roles of both CNOT1 and CCR4-NOT in development and disease.

In this study we aimed to provide evidence supporting the *in silico* predictions of two CDH patient variants, and additionally to examine the specific roles of these genes in normal diaphragm development. We generated two germline mouse models harboring orthologous patient-specific variants, which represent two distinct forms of CDH and provide further evidence regarding CDH etiology. Given the limited understanding of the genetic basis of CDH, our study not only validates the contribution of these variants and genes for CDH but also the investigates the novel molecular pathways and downstream regulation essential for normal diaphragm development.

## Results

The patient variants modeled were discovered through genome sequencing of a cohort of 827 CDH patient-parent trios [5, 16]. Both patients are male, unrelated, and presented with an isolated left, dorsal congenital diaphragmatic hernia with no history of pulmonary hypertension or pulmonary insufficiency. Both variants, c.3536C>T p.T1179M *CDC42BPB* and c.1867C>T p.R623W *CNOT1*, arose *de novo* and have CADD scores of 29 and 27, respectively. The patient with the *de novo CDC42BPB* variant also showed normal neurodevelopmental outcome with a Vineland Adaptive Behavioral Scale score of 95, with assessment taking place given previous association of *CDC42BPB* damaging variants with neurodevelopmental disorders [22]. The patient with the *de novo CNOT1* variant had no other reported phenotypes. Neither gene has previously been implicated in CDH or diaphragm development.

At the time of patient variant discovery, a knockout allele of *Cdc42bpb* generated by targeted deletion of the second exon (the first full coding exon) was under investigation as part of the Knockout Mouse Phenotyping Program [35] at The Jackson Laboratory (C57BL6NJ-*Cdc42bpb^em1(IMPC)J^*/J;MGI:5812907; homozygous KO referred to as *Cdc42bpb^−/−^*; Fig. 1A). Intercrosses of heterozygotes to generate homozygous cohorts revealed a high rate of pre-wean (prior to 3 weeks of life) lethality (Fig. 1B), with only 2/604 weaned animals having two copies of the exon 2 deletion. Although we also noted fewer than expected heterozygous animals (359/604, 401 expected, χ^2^  *P*-value < 0.001), we observed no morphology differences between wildtype and heterozygotes and thus focused our analysis on homozygote embryos. We further characterized the sub-viability finding using the KOMP2 embryonic phenotyping pipeline. From initial phenotyping, *Cdc42bpb^−/−^* leads to overall smaller embryo size and pallor (Fig. 1C; additional observations available on the IMPC portal: mousephenotyping.org/data/genes/MGI:2136459). Deletion of exon 2 was confirmed to lead to loss of protein expression via western blot using protein isolated from E15.5 diaphragms of wildtype and homozygous embryos (Fig. 1D).

**Figure 1:**
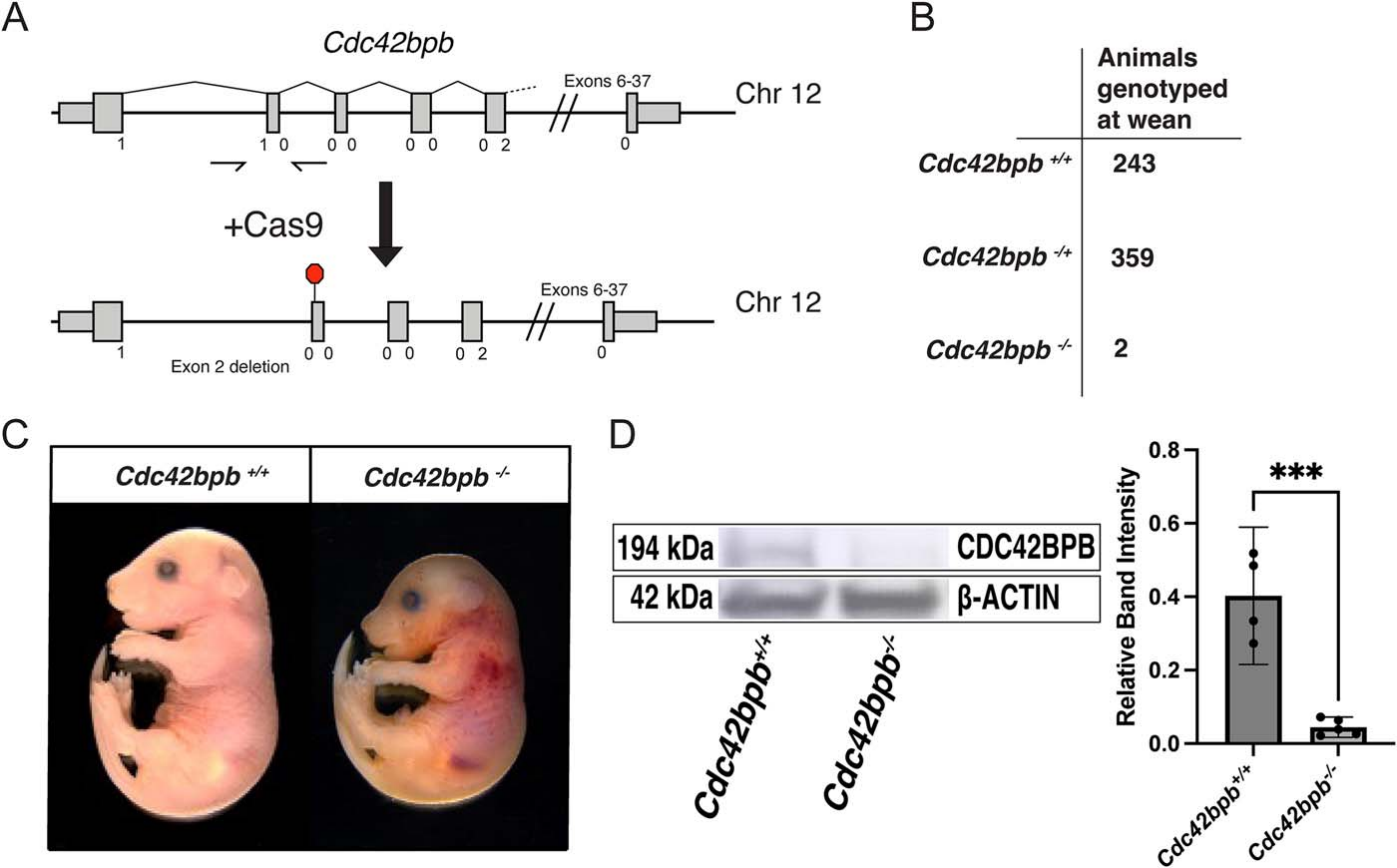
Homozygous deletion of *Cdc42bpb* exon 2 leads to pre-wean lethality. A) Targeted deletion of the second exon results in a reading frame shift and a premature stop codon at the tenth amino acid of exon 3. B) Table of genotyped mice at wean. C) Representative images of E18.5 embryos, showing smaller size and minor hemorrhage in the mutant sample. D) CDC42BPB protein expression is significantly reduced in *Cdc42bpb^−/−^* E15.5 embryonic diaphragms (N = 5) compared to wild-type controls (N = 4). ^***^ = < 0.001 by Student’s *t-*test. Error bars represent 95% confidence intervals.

To assess for CDH, we performed a detailed assessment of internal organ structure using iodine-contrast micro-computed tomography (μCT) scan data generated as part of the KOMP2 screen at E18.5, supplemented by scans at additional time points at E17.5 and postnatal day 1 (P1). From the coronal sections of scanned embryos, incomplete diaphragm closure and minor eventration of the liver were discovered in *Cdc42bpb^−/−^* embryos compared to *Cdc42bpb^+/+^* littermates, as highlighted by the dashed outlines in Fig. 2. Three-dimensional segmentation and rendering of the developing diaphragm using ITK-SNAP [36] revealed that the herniation is consistently located ventrally, spanning from the ventral midline to the central tendon with varying degrees of muscle loss (Fig. 2, Supplemental Fig. 1). The distance between the most ventral edges of the patterned muscle is significantly increased in *Cdc42bpb^−/−^* as compared to both *Cdc42bpb^−/+^* and *Cdc42bpb^+/+^* diaphragms (Fig. 2C), demonstrating that this diaphragm phenotype is severe and highly penetrant, but that one copy of *Cdc42bpb* is sufficient for normal diaphragm development. Although this does not phenocopy the human patient’s dorsally located diaphragmatic hernia, it does demonstrate that *Cdc42bpb* plays a critical role in proper diaphragm development.

**Figure 2:**
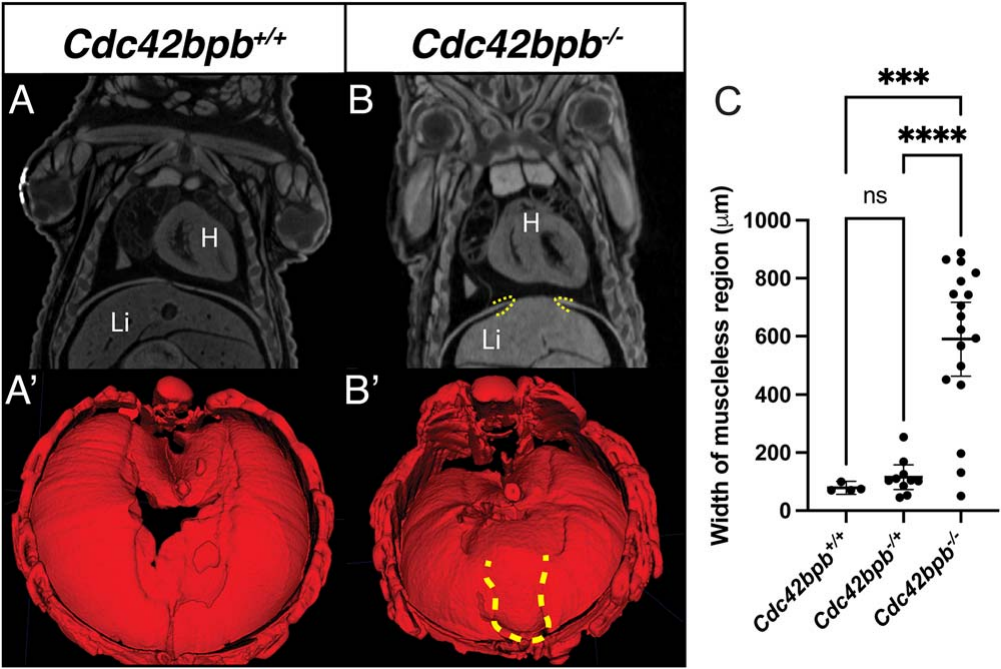
*Cdc42bpb* mutants show ventral diaphragm herniation. A) Whole body μCT scanning reveals loss of ventral diaphragm muscle (border of muscleless region highlighted with dashed-line). Under this muscleless region the liver is beginning to herniate. C) Quantitation of the distance between left and right costal muscle sides. H- heart, Li- liver, ^^***^^ = < 0.001, ^^****^^ = < 0.00001 by one-way ANOVA. Error bars represent 95% confidence intervals.

The region of the diaphragm altered in *Cdc42bpb^−/−^* is the last to be patterned during development [8, 9]. As the ventral midline is the last region where myofibers form (around E16.5), there is often a small ‘gap’ at the midline between the left and right costal muscle groups. *Cdc42bpb^−/−^* embryos consistently have a significantly greater distance between the left and right muscle groups compared with both *Cdc42bpb^−/+^* and *Cdc42bpb^+/+^* at the same embryonic stage (Fig. 3). To determine whether this defect in muscle patterning is due to developmental delay, diaphragms from E14.5 to E16.5 were dissected and developing muscle fibers were visualized colorimetrically with alkaline phosphatase staining against Myosin (Skeletal, fast) protein. As early as E14.5 there is a significant distance from the ventral leading edges between the migrating developing muscle groups on the left and right side of the embryo, suggesting that the muscle pattern is either delayed or altered throughout diaphragm development (Fig. 3).

**Figure 3:**
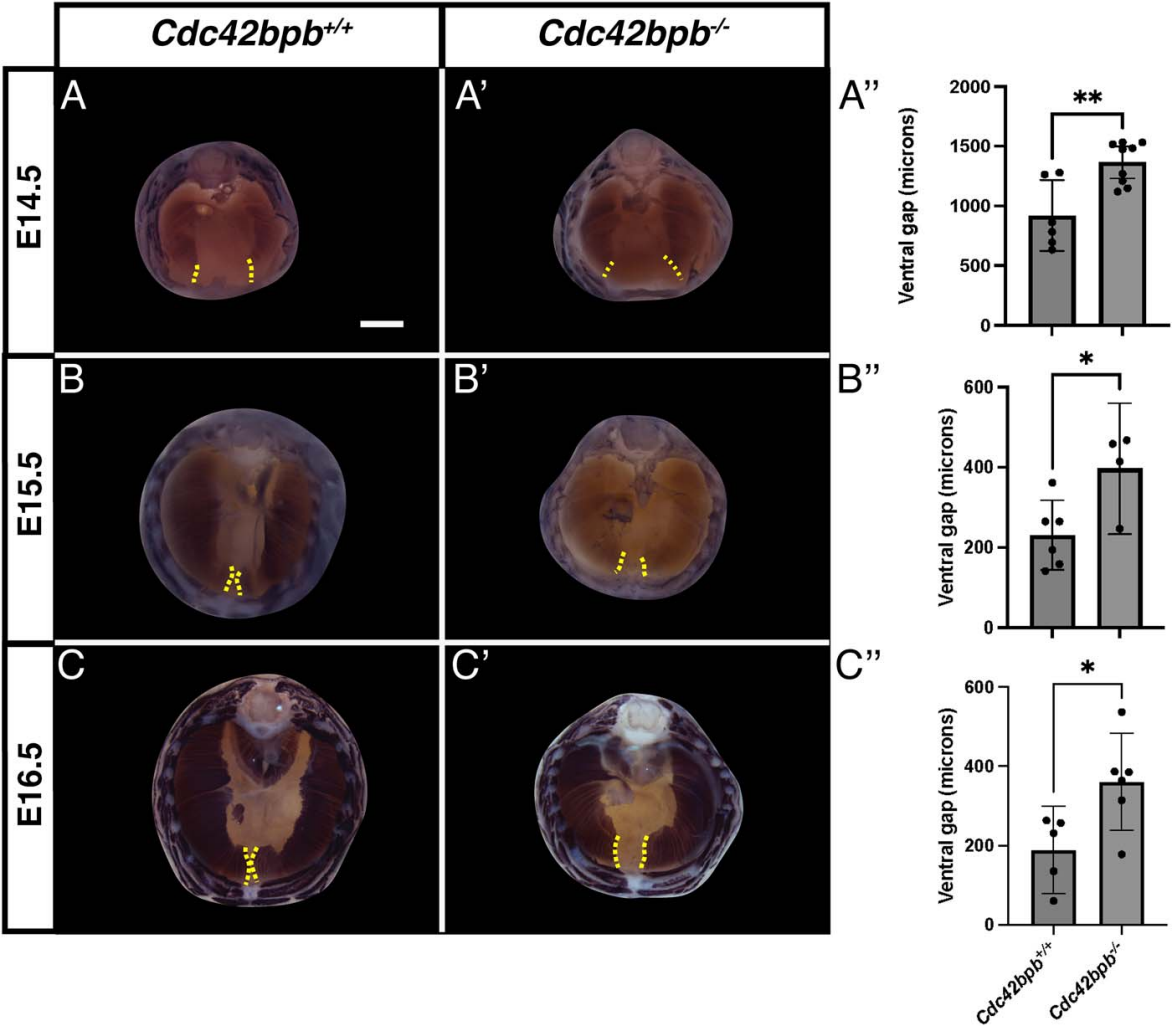
Developmental timecourse of diaphragm formation in *Cdc42bpb*^−/−^ embryos. Dissected diaphragms stained for skeletal myosin protein at A) E14.5, B) E15.5 and C) E16.5. *Cdc42bpb^+/+^* representative images in left column, *Cdc42bpb^−/−^* representative images in right column showing differences of ventral muscle patterning and distance between left and right ventral muscle edges. Edge of patterned muscle fibers highlighted by dashed line. Ventral gaps were measured across the midpoint of patterned muscle and quantified. ^*^ = < 0.05, ^**^ = < 0.01 by Student’s *t-*test. Scale bar- 1000 μm. Error bars represent 95% confidence intervals.

Although highly penetrant, the *Cdc42bpb^−/−^* diaphragm ventral defect is not predicted to cause embryonic lethality, and ventral defects often present as sub-clinical in humans [3]. To determine a potential driver of the neonatal lethality, a thorough manual assessment of internal structures revealed clear ventricular septation defects (VSDs) in 9/9 *Cdc42bpb^−/−^* E18.5 embryos examined (Fig. 4). *Cdc42bpb^−/−^* neonates died during postnatal day 1, significantly earlier than *Cdc42bpb^−/+^* and *Cdc42bpb^+/+^* littermates. The small number of incidental deaths of *Cdc42bpb^−/+^* and *Cdc42bpb^+/+^* littermates were likely due to stress from handling (Fig. 4D). Along with VSDs, lung gross morphology differences were also detected from *Cdc42bpb^−/−^* μCT scans. From 3D rendering of scanned images,16/18 lungs present with the distal edges and accessory lobe horn appearing blunted, though overall lung volume is unchanged between *Cdc42bpb^+/+^* and *Cdc42bpb^−/−^* animals (Supplemental Fig. 2A and B’, yellow dashed lines). The dysmorphology of E18.5 lungs suggested minor patterning defects during mid-gestation. Bronchial development was visualized using whole mount immunostaining against cytokeratin in isolated lungs from E13.5 and E14.5. As the E18.5 phenotype appears to impact all lobes, for ease of dissection the left lobe was used for analysis at the mid-gestation timepoint (Supplemental Fig. 2D and E). Counting of the distal lung buds at each stage revealed no significant differences (Supplemental Fig. 2F). This suggests that the apparent later-stage dysmorphology may be due to a more subtle disruption of lung maturation at later time points.

**Figure 4:**
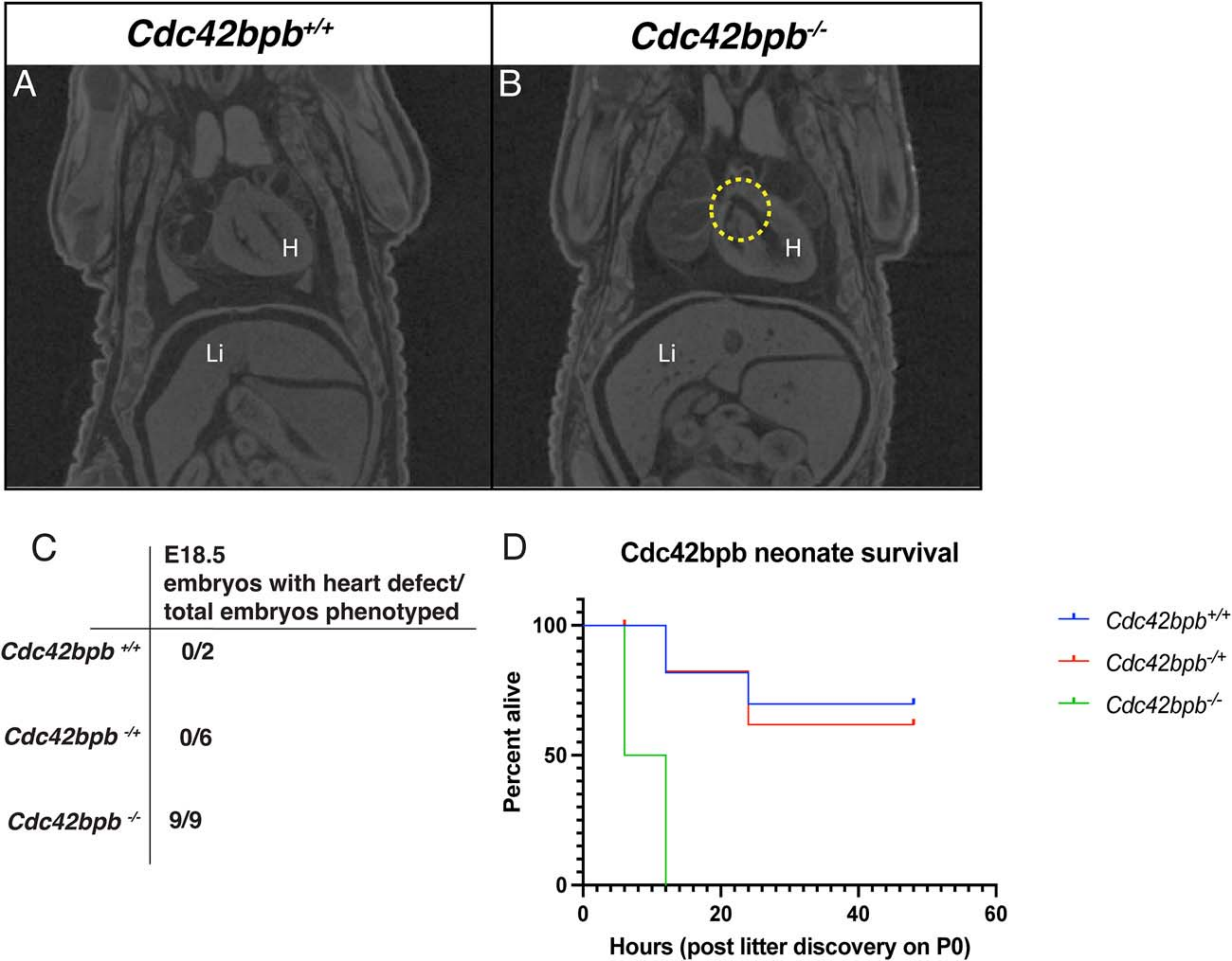
Heart defects and perinatal lethality in *Cdc42bpb^−/−^* mutants. Coronal μCT sections of A) *Cdc42bpb^+/+^* and B) *Cdc42bpb^−/−^* highlighting the septation defect (dashed line). C) Number of samples with observable septation defects by genotype. D) Neonate survival plot, with hour 0 set as when litter was discovered on postnatal day 0.

To determine the cell populations impacted, the lungs of E18.5 and P0 *Cdc42bpb^−/−^* were assessed for pulmonary hypoplasia phenotype which may coincide with the diaphragmatic defects. *Cdc42bpb^+/+^* and *Cdc42bpb^−/−^* mouse lungs appeared morphologically similar by hematoxylin and eosin (H&E) staining (Supplemental Fig. 3A). Through staining of HOPX (Homeodomain-only Protein Homeobox), which marks the alveolar type I (ATI) cells responsible for gas exchange, E18.5 *Cdc42bpb^−/−^* embryos exhibited a reduction in ATI populations (Supplemental Fig. 3B and C). Despite this alveolar differentiation defect in E18.5 *Cdc42bpb^−/−^* embryos, the P0 mutants did not exhibit a reduction in ATI cells and instead showed a recovery of the ATI population (Supplemental Fig. 3D). Staining for SPC (Surfactant Protein-C), a marker for the alveolar type II (ATII) cells which are required for surfactant production, revealed no population differences (Supplemental Fig. 3E). Transitional ATI/ATII populations were similar between control and mutant mice (Supplemental Fig. 3F). Airway epithelial populations such as club cells, marked by CC10+ cells, showed no quantitative difference (Supplemental Fig. 3G). The pulmonary vasculature was assessed via α-SMA and VWF staining, respectively marking the smooth muscle wall and the endothelial cells, but there was no difference in the vascular wall thickness between *Cdc42bpb^+/+^* and *Cdc42bpb^−/−^* mice (Supplemental Fig. 3H). These data suggest that *Cdc42bpb* plays a minor role in lung development and function, and loss of the gene does not impact respiration.

Phenotyping of the KOMP deletion mouse line demonstrates a role of *Cdc42bpb* in diaphragm development; however, the impact of the patient missense variant, c.3536 C>T p.T1179M is unclear. To determine the effect of this variant, we employed CRISPR/Cas9 editing, and homology-directed repair (HDR) to create the orthologous C>T transition in mouse zygotes and directly characterized founder (F0) embryos [37, 38] (see Methods). Edited E17.5 and E18.5 embryos were harvested, genotyped, and scored for diaphragm defects using μCT whole embryo scanning (Fig. 5). Genotypes were inferred from Sanger trace data using Inference of CRISPR Edits (ICE) software (ICE CRISPR Analysis. 2025. v3.0. EditCo Bio) and scored for successful installation of the target base (Knock-in Score) or creation of an insertion or deletion (indel) leading to frameshift and a predicted gene knockout (Knockout Score) (See Supplemental Table 1 for ICE generated scores). Embryos with one (20–70 Knock-in Score) or two (70–100 Knock-in Score) copies of the patient variant presented with minimal ventral diaphragm gap distances, whereas embryos with both copies of *Cdc42bpb* predicted to be lost (70–100 Knockout Score) presented with ventral gaps similar to the *Cdc42bpb^−/−^* associated phenotype (Fig. 5). Plotting each ICE score (Knockout and Knock-in) against ventral gap measurement does reveal a positive correlation of Knockout Score amount and herniation (Supplemental Fig. 4A). Conversely there is a minor negative correlation of ventral gap with Knock-in Score amount (Supplemental Fig. 4B), though it should also be noted all gap measurements are larger than unedited embryos measured (denoted by #). These data support the assertion that any bi-allelic loss of *Cdc42bpb* leads to a diaphragm defect but do not validate the patient variant as directly causative for CDH.

**Figure 5:**
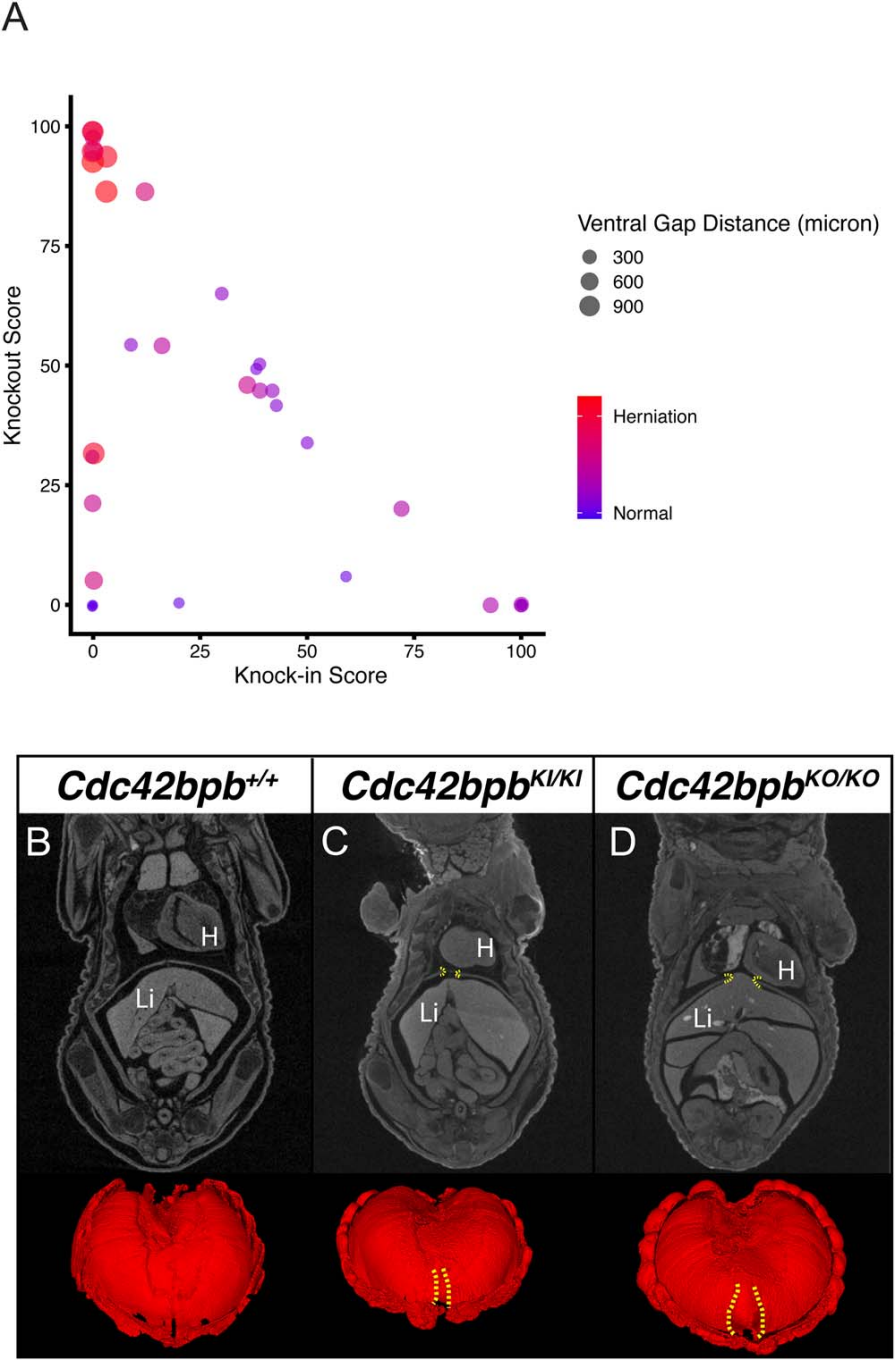
Functional validation of the *CDC42BPB*-T1179M variant for CDH in F0 mouse embryos. A) Scatterplot comparing ICE (see methods) knock-in score (specific variant; x-axis) and knockout score (frame-shifting indel; y-axis) scores for each scanned embryo (datapoints). Herniation status of E18.5 embryo denoted by datapoint color, and ventral gap distance denoted by size of datapoint. B), C), and D) representative coronal sections and diaphragm renders for unedited, 100% knock-in and 100% knockout embryos, respectively. Ventral gaps in both section and render highlighted by yellow dash lines. H- heart, Li- liver.

The second candidate variant in this study was a predicted damaging missense variant in *CNOT1*, a critical gene in the CCR4-NOT complex not previously associated with CDH*. Cnot1* is a cell-essential gene, with complete loss leading to embryonic lethality in mice [26]. To determine whether the R623W mutation causes CDH, we engineered the orthologous C>T variant using CRISPR/Cas9 mediated editing and established a germline model, C57BL/6NJ-*Cnot1^em1Murr^*/Murr (referred to as *Cnot1^R623W^*; see Fig. 6A and Methods). Both heterozygous and homozygous animals are viable and fertile and show no significant change in expected Mendelian ratios of genotypes (*Cnot1^+/+^* 77/351, *Cnot1^+/R623W^* 189/351 and *Cnot1^R623W/R623W^* 85/351). Late-stage embryos and early-life neonates (E18.5-P2) across multiple generations were collected and either μCT imaged or dissected to score diaphragm defects (Fig. 6B–E).

**Figure 6:**
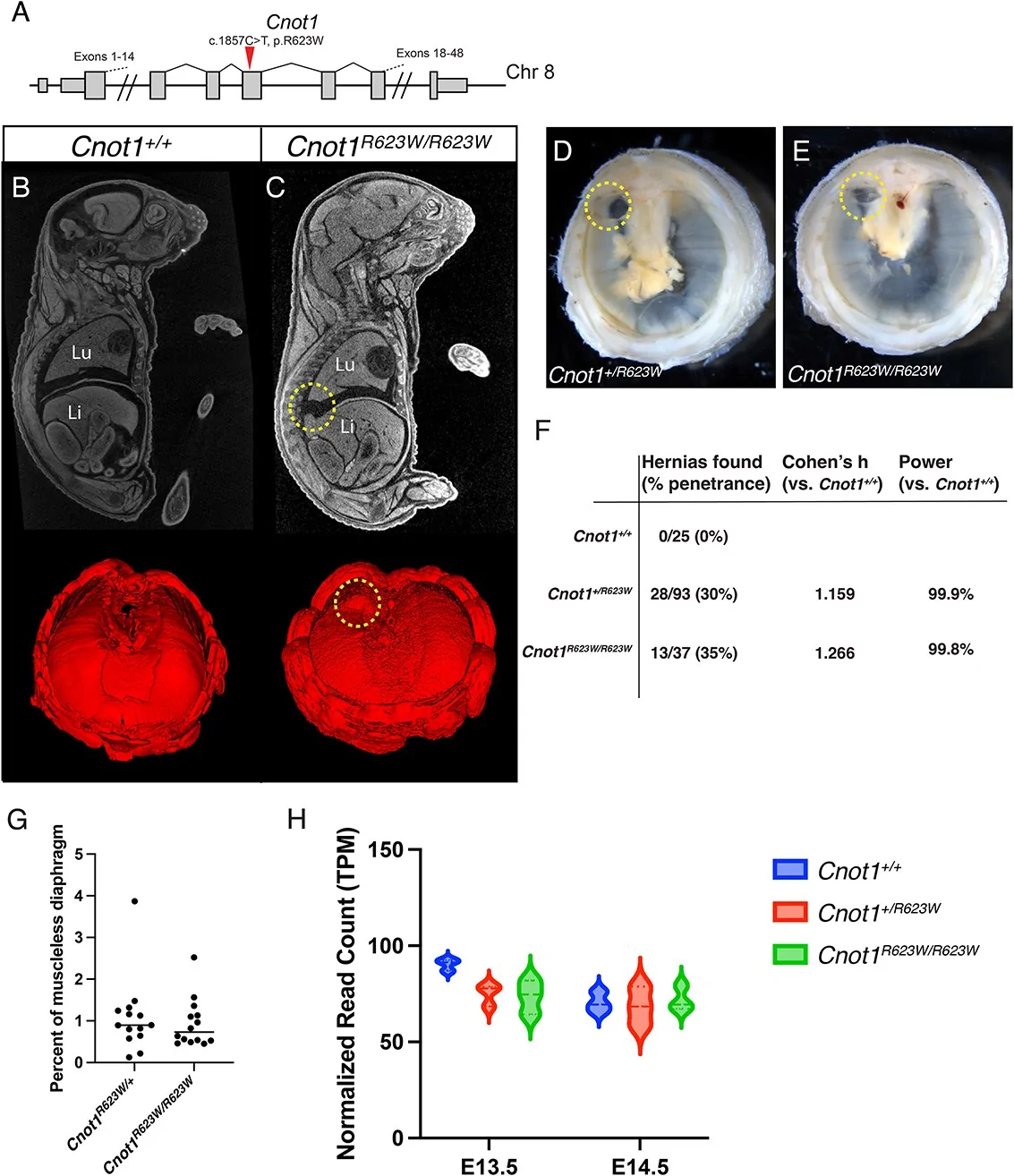
The *CNOT1*-R623W patient-specific variant leads to dorsal diaphragmatic hernia with incomplete penetrance in both heterozygous and homozygous mouse embryos. A) Location of the patient-specific missense variant inserted into exon 17 of mouse *Cnot1*. Representative images of B) *Cnot1^+/+^* and C) *Cnot1^R623W/R623W^* of μCT scanned E18.5 embryos and rendered diaphragms from scans. Dorsal diaphragmatic hernia in *Cnot1^R623W/R623W^* highlighted by dashed lines. Dissected P1 diaphragms, with livers removed, with dashed lines highlighting dorsal herniations present in D) *Cnot1^+/R623W^* and E) *Cnot1^R623W/R623W^*. Total number of diaphragmatic hernias discovered in E17.5-P1 animals across all genotypes, with percent penetrance noted. Post-hoc power analysis using Cohen’s h and corresponding power (α = 0.05) included. G) Quantitation of muscleless regions (area of herniated diaphragm/area of total diaphragm muscle) between *Cnot1^+/R623W^* and *Cnot1^R623W/R623W^*. Not significant by Student’s *t-*test. H) RNA expression of *Cnot1* from bulk RNA sequencing from E13.5 and E14.5 diaphragms (N = 3 per genotype). No significant difference was found in normalized read count (transcript per million or TPM) by one-way ANOVA.

Dorsally located amuscular regions were discovered in animals with either one or two copies of the R623W allele, at similar low penetrance (Fig. 6F). The relative size of the defect is also similar between heterozygous and homozygous animals that present with a diaphragm defect (Fig. 6G). From bulk RNA-sequencing of E13.5 and E14.5 diaphragms no significant change in expression between genotypes was found (Fig. 6H, N = 3 diaphragms per genotype per stage). Compared to the ventrally located defect found in the *Cdc42bpb^−/−^*, the *Cnot1^R623W^* phenocopies the dorsally located diaphragm defect reported in the human patient. As found in human patients [1, 3], these dorsally located hernias are more likely to lead to a compression of the developing lungs, as seen in the highlighted region of the μCT sagittal section in Fig. 6C. While phenocopying the patient form of CDH, the diaphragm defect appears sublethal due to low penetrance, given the minimal impact on lethality at wean.

*Cnot1* encodes a critical component of the CCR4-NOT protein complex, which regulates mRNA processing by deadenylation of the poly(A) tail [23, 25], and thus we hypothesized that the patient’s variant impacts CCR4-NOT complex function and global gene expression. To test this, developing diaphragms were collected at E13.5 and E14.5 from *Cnot1^+/R623W^*, *Cnot1^R623W/R623W^* and wildtype littermates, total RNA was isolated and RNA sequenced (see Methods). At E13.5 gene expression was not significantly changed when comparing wildtype to *Cnot1^+/R623W^* or *Cnot1^R623W/R623W^* samples (Supplemental Fig. 5, Supplemental Table 2). At E14.5 a minimal number of genes are differentially expressed comparing either *Cnot1^+/R623W^* or *Cnot1^R623W/R623W^* to wildtype (Fig. 7A and D). When analyzed with STRING [39] an interaction network forms between genes differentially expressed in *Cnot1^+/R623W^* compared to *Cnot1^+/+^* (Fig. 7B and C), and the differentially expressed genes are enriched for ‘abnormal morphology’ and ‘lethality’ Mammalian Phenotype Ontology (MPO) [40] terms (Supplemental Table 3) from the Mouse Genome Database (MGD) [41]. When comparing *Cnot1^R623W/R623W^* and wildtype, only 7 genes were found to be differentially expressed, and no enrichment terms were found when analyzed with STRING.

**Figure 7:**
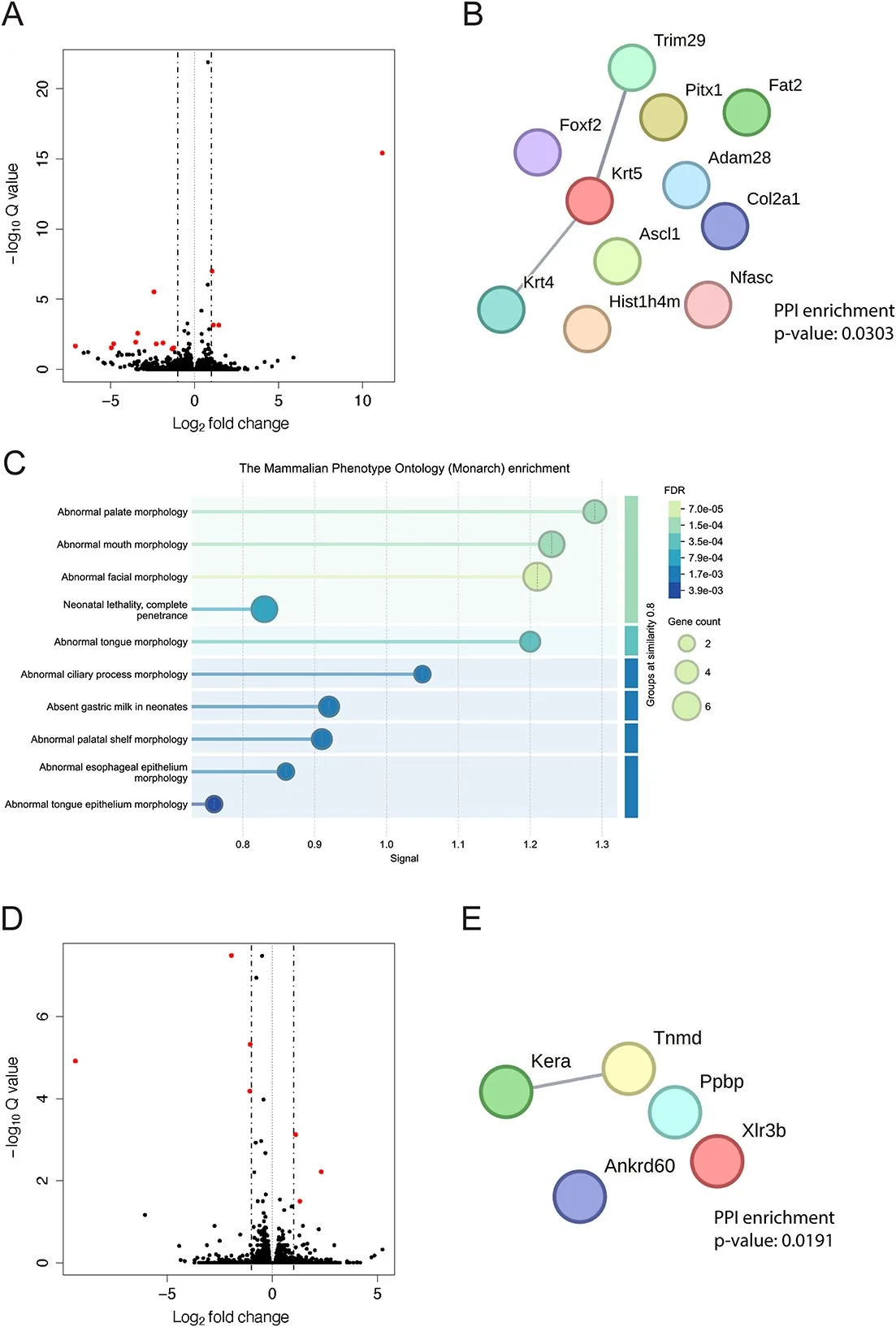
Transcriptional changes in *Cnot1* mutant diaphragms at E14.5. Volcano plots highlighting differential expressed genes in comparisons of: A) *Cnot1^+/+^* vs. *Cnot1^+/R623W^* and D) *Cnot1^+/+^* vs *Cnot1^R623W/R623W^*. Red datapoints represent genes with absolute log_2_ fold change greater than 1 and p-adjusted (Q) value less than or equal to 0.05. STRING network analysis of significant differentially expressed genes for B) *Cnot1^+/+^* vs. *Cnot1^+/R623W^* and E) *Cnot1^+/+^* vs *Cnot1^R623W/R623W^*. Lines represent genes with known interaction. C) STRING ontology enrichment of *Cnot1^+/+^* vs. *Cnot1^+/R623W^* differentially expressed genes, highlighting monarch terms significantly enriched. No enrichment terms were found for *Cnot1^+/+^* vs *Cnot1^R623W/R623W^*.

Given that *Cnot1* is critical in post-transcriptional mRNA processing, we also investigated changes in transcript isoforms in samples with the *Cnot1^R623W^*. Comparison of either *Cnot1^+/R623W^* or *Cnot1^R623W/R623W^* transcript isoform expression levels to *Cnot1^+/+^* revealed many differentially expressed transcripts (DETs) at both E13.5 (Supplemental Fig. 6) and E14.5 (Supplemental Fig. 7, Supplemental Table 4). STRING network analysis revealed an enrichment of related DETs only at E14.5 comparisons, with protein–protein interaction (PPI) enrichment p-values of 0.00241 for *Cnot1^+/+^* x *Cnot1^+/R623W^* and 0.0242 for *Cnot1^+/+^* x *Cnot1^R623W/R623W^* compared to p-values of 0.496 and 0.107 for equivalent comparisons at E13.5. From functional enrichment term analysis of DETs, many terms across STRING databases were found (Supplemental Table 5), varying across each comparison. Across all comparisons only the UniProt annotated keywords ‘Alternative splicing’ and ‘Phosphoprotein’ were consistently significantly enriched (Supplemental Figs 6C–F and 7C–F). ‘Alternative splicing’ is likely in part due to the comparison of distinct gene isoforms (created by mRNA splicing) being compared. An enrichment of phosphorylated proteins may be more biologically relevant, though this keyword encompasses many proteins in the mouse genome (7592 proteins within the STRING Uniprot database). Analyzing significant DETs shared across the two comparisons at each stage (17 DETs at E13.5 and 22 at E14.5, Figs 8 and 9) did reveal that the ‘Phosphoprotein’ keyword remains enriched at E13.5 (with 14/17 proteins tagged, Fig. 8D red highlighted proteins), though only ‘Bromo domain’ (3/22 proteins) remains at E14.5 (Fig. 9D, red highlighted proteins).

**Figure 8:**
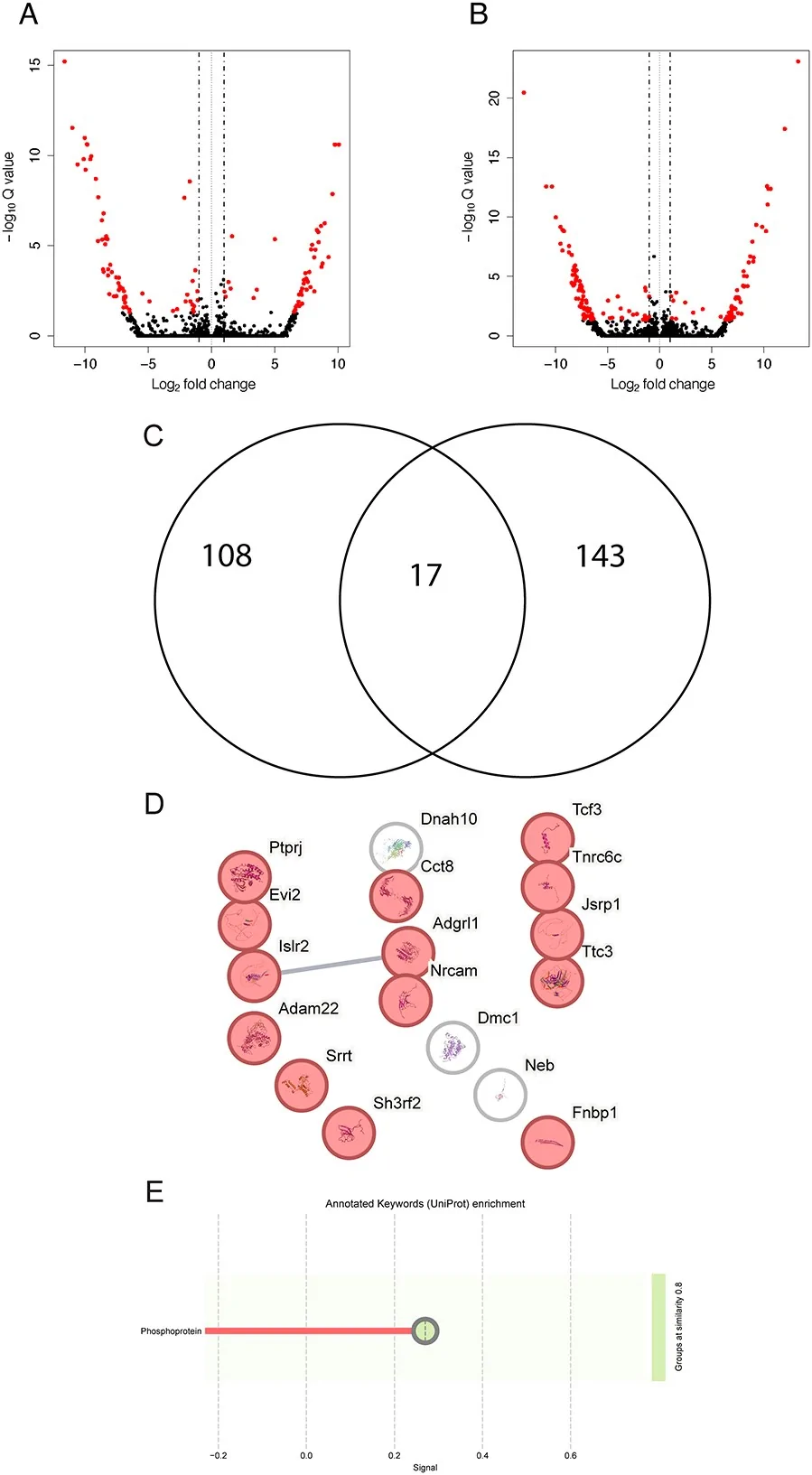
Differentially expressed transcription isoforms in *Cnot1* mutants at E13.5. Volcano plots of transcript differential analysis between A) *Cnot1^+/+^* vs. *Cnot1^+/R623W^* and B) *Cnot1^+/+^* vs. *Cnot1^R623W/R623W^*. Transcripts (datapoints) with absolute log_2_ fold change greater than 1 and p-adjusted (Q) values less than 0.05 highlighted in red. C) Number of significantly expressed transcripts unique and shared between *Cnot1^+/+^* vs. *Cnot1^+/R623W^* and *Cnot1^+/+^* vs. *Cnot1^R623W/R623W^* comparisons. D) STRING analysis on transcripts shared between differential comparisons. E) STRING ontology analysis enriched term within shared significant transcripts. Genes within the enriched term highlighted in red in D).

**Figure 9:**
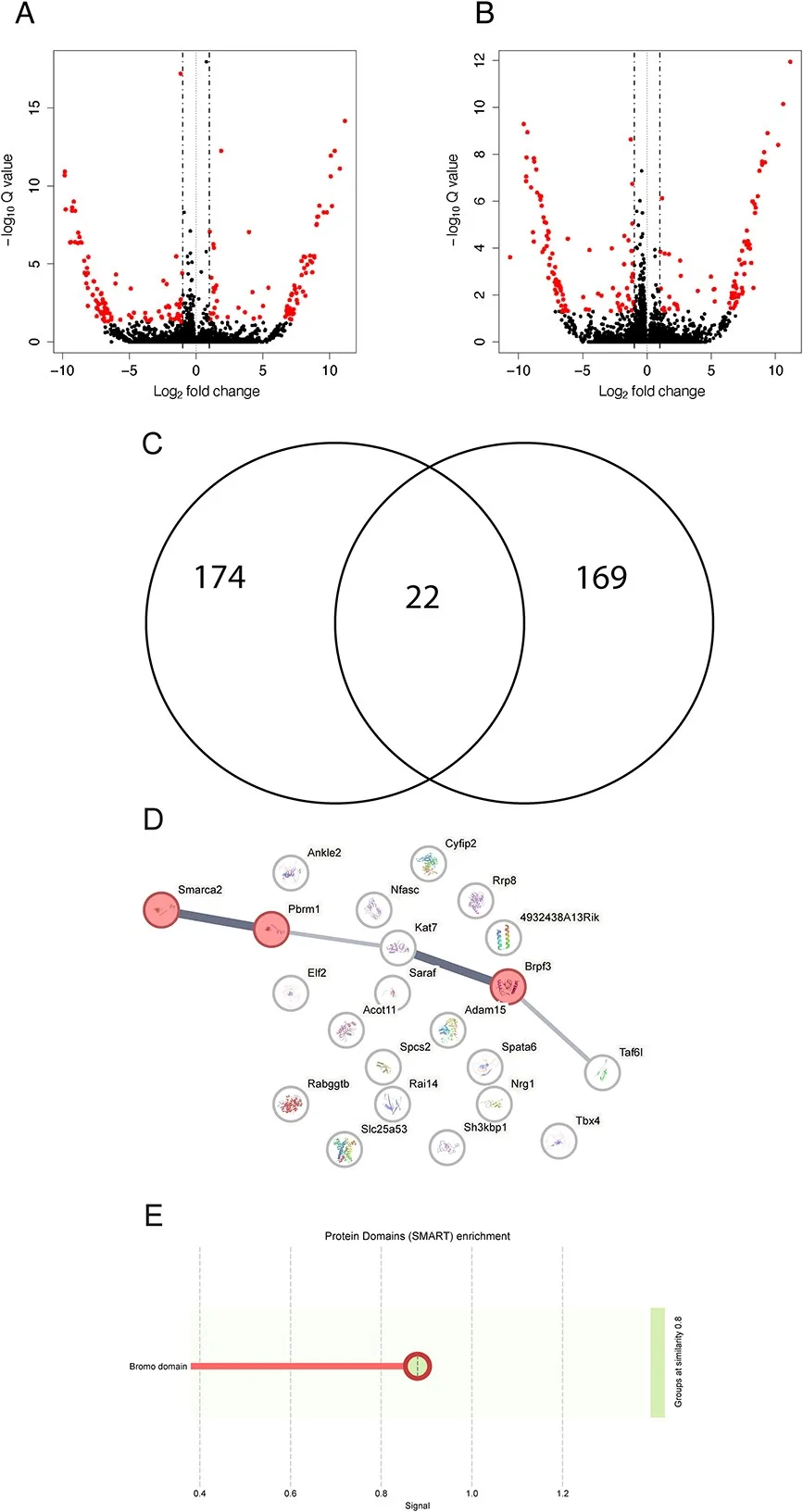
Differentially expressed transcription isoforms shared in in *Cnot1* mutants at E14.5. Volcano plots of transcript differential analysis between A) *Cnot1^+/+^* vs. *Cnot1^+/R623W^* and B) *Cnot1^+/+^* vs. *Cnot1^R623W/R623W^*. Transcripts (datapoints) with absolute log_2_ fold change greater than 1 and p-adjusted values less than 0.05 highlighted in red. C) Number of significantly expressed transcripts unique and shared between *Cnot1^+/+^* vs. *Cnot1^+/R623W^* and *Cnot1^+/+^* vs. *Cnot1^R623W/R623W^* comparisons. D) STRING analysis on transcripts shared between differential comparisons. E) STRING ontology analysis enriched term within shared significant transcripts. Genes within the enriched term highlighted in red in D).

Conditional loss of *Cnot1* has been shown to lead to global changes in polyadenylation, and the patient predicted damaging variant is located near the region where CNOT7 and CNOT8, key proteins in the adenylation regulation, bind [23, 27]. To determine if a change in polyadenylation was captured in the total RNA sequenced diaphragm samples, the tool APAlyzer [42] was used. APAlyzer uses reference polyadenylation sites (PAS) databases to identify all polyA sites across the region, then compares read density across mutant and control samples at those sites for relative expression scores, allowing for differential polyadenylation analysis at the gene level [42]. Though APAlyzer found genes with signatures of polyadenylation change when wildtype samples were compared to *Cnot1^+/R623W^* and *Cnot1^R623W/R623W^* samples at both developmental stages (Supplemental Fig. 8A–D), no gene reached significance after multiple testing correction (Supplemental Fig. 8A’–D’, Supplemental Table 6). Polyadenylation sites of the top ten genes identified to have altered polyadenylation were found, upon visual inspection, to have minimal to no change in read density across samples in the Integrative Genome Viewer (IGV) [43]. Overall, there appears to be little to no detectable change in polyadenylation, although due to the low penetrance of the CDH phenotypes and potential hypomorphic nature of the *Cnot1^R623W^* allele, any change in polyadenylation or overall gene expression may be difficult to detect.

## Discussion

As clinical genomic sequencing efforts and technologies expand, the number of novel variants and genes predicted to underlie disease including congenital anomalies will continue to increase. In parallel, methods to validate a gene or variant’s role in disease pathogenesis are vital. Here we present *in vivo* characterization validating the role of two novel CDH variants predicted to drive severe forms of CDH in human patients. Our models were useful for interrogating the roles of both the gene and the patient-specific variant identified. For *Cdc42bpb,* we leveraged an existing knockout resource to establish that the gene is necessary for diaphragm development. In contrast, through F0 modeling via direct phenotyping of Cas9-edited embryos we were not able to validate that the patient-specific missense variant leads to diaphragm defects. However, loss-of-function Cas9-mediated frameshift mutations lead to ventral hernias identical to those identified in our germline knockout model. The consistent ventral hernia phenotype seen in mice does not phenocopy the dorsal hernia seen in patients but does support a role for the gene in normal diaphragm development. Further work is needed to understand this discrepancy, which has been found in other mouse CDH models [44], and how much this phenomenon reflects species-specific differences at the genetic or developmental level. For *Cnot1,* our patient-specific missense allele resulted in dorsal, isolated CDH in both heterozygous and homozygous samples, phenocopying the patient at both zygosities, consistent with the *de novo* mutation effect. With these two mouse lines we can model two ends of the spectrum of CDH severity as determined by diaphragm defect size and location.

The *Cnot1^R623W^* allele leads to dorsal diaphragm hernias and has a minor impact on RNA transcription. Though phenocopying the Bochdalek (dorsal) hernia found in the human patient carrying this allele, the missense mutation did not lead to detectable lethality in either heterozygous or homozygous mice. The incomplete penetrance may lead to rare instances of lethality, which were not detected in our analysis, or alternatively the herniation is not severe enough to impact animal fitness. To date no adult mice with either one or two copies of *Cnot1^R623W^* have been found to have a diaphragmatic hernia similar to those found in embryos or neonates. However, our findings support a role for *Cnot1* in diaphragm development and corroborate the patient missense mutation as likely contributing to CDH. Bulk RNA sequencing analysis showed two recurrent Gene Ontology (GO) enrichment terms: ‘Phosphoprotein’ and ‘Alternative Splicing’, the latter consistent with a splicing role for the complex. Although significant across all four comparisons, both terms have a lower enrichment effect, possibly due to the large number of genes in the ‘Phosphoprotein’ and ‘Alternative Splicing’ networks in the Uniprot database [45]. Previously it has been shown that depletion of CNOT1 *in vitro* correlates with altered phosphorylation of critical cell cycle components leading to senescence [46]. Recently, it has also been shown that phosphorylation changes of CCR4-NOT binding partners alter the amount of deadenylation of target mRNAs [47]. It is likely that the *Cnot1^R623W^* allele is hypomorphic, leading to more subtle changes compared to full depletion of *Cnot1*. Although we hypothesized that the deadenylation function may be altered enough in the developing diaphragm to lead to herniation, as this is a well-documented and critical function of the CCR4-NOT complex [24, 25], we did not detect a significant impact on mRNA 3′ adenylation in diaphragms with one or two copies of *Cnot1^R623W^*. This leaves at least three possibilities to be tested: 1) we are underpowered to detect a low level of alternative polyadenylation (APA) differences in discrete transcripts in the diaphragm, consistent with variable severity and incomplete penetrance in *Cnot1^R623W^* mutants; 2) the threshold of altered adenylation in the diaphragm is so sensitive that the stochastic differences across animals are enough for some to develop hernias, for which again we are underpowered to detect; or 3) the herniation associated with *Cnot1^R623W^* is not driven by APA changes in CCR4-NOT complex function. Further investigation to distinguish these mechanisms is necessary. Given that the germline knockout of *Cnot1* is embryonic lethal, conditional analysis in diaphragm-specific cell types will be critical for future mechanistic studies into the role of *Cnot1* in diaphragm development.

Homozygous loss of *Cdc42bpb* leads to large ventral gaps in the diaphragm with high penetrance, minor changes in lung morphology, and lethal heart septation defects. Similar ventral defects have been reported in other mouse models of cytoskeletal genes including a *Pls3* missense mutation and a *Myh10* knockout [44, 48]. However, in both *Pls3* and *Myh10* models, ventral body wall defects were reported, which to date have not been found in *Cdc42bpb^−/−^* mice. Ventral diaphragm muscle mis-patterning was also seen when *HGF*/*MET* signaling was disrupted, both genetically and pharmacologically, in the mouse embryonic diaphragm [14]. The *HGF/MET* signaling pathway is critical for muscle recruitment from the somites to the PPFs, and the authors demonstrated a loss of myogenic cells at the leading edge of muscle patterning at E15.5. This could suggest a similar loss of myogenic cells in *Cdc42bpb^−/−^* diaphragms, leading to a similar ventral gap.

For *Cdc42bpb* and other CDH mouse models that do not phenocopy patient dorsal hernias, it is plausible that the individual genetic context, combined with environmental exposures and stochastic variables that are not captured in our isogenic mouse model, lead to the emergence of a clinically relevant CDH. Additional approaches, including the use of alternative mouse genetic backgrounds or including teratogenic exposures, could result in dorsal hernias that more closely recapitulate those seen in patients. Despite these limitations, our study provides a foundation for unraveling the complex etiology of CDH by advancing our understanding of the critical genes necessary correct diaphragm and lung formation. Given the high level of penetrance of the ventral diaphragm defect in *Cdc42bpb^−/−^* embryos this model provides a potential platform for further investigation into complex genetics of CDH development.

To date no *Cdc42bpb^−/−^* neonate has any noted respiration phenotypes, though the observation window is small; thus, it is likely these noted phenotypes at E18.5 are not impacting overall lung function. However, given the difference in diaphragm phenotype between the human patient and *Cdc42bpb^−/−^* embryos gene-disrupting variants in human *CDC42BPB* may lead to more severe lung defects. *Cdc42bpb^−/−^* neonate lethality is likely driven by the structural heart defects found through μCT imaging (Fig. 4) and not by defects in the diaphragm or lungs. This co-morbidity also reflects the common presentation of CHD with CDH in human patients [1], and cardiovascular defects in patients with *CDC42BPB* variants have been reported [22]. This pleiotropy across heart, lung and diaphragm is found across many CDH-related genes, both in human patients presenting with CDH, CHD or both and in *in vivo* animal modeling [1, 15]. Other pleiotropic CDH genes such as *Gata4* and *Zfpm2* lead to early embryonic lethality when deleted in the mouse germline [49–51], before diaphragm development begins, unlike *Cdc42bpb* deletion, which leads to a cardiac septation defect impacting neonate fitness, but not early embryonic development. Further investigation into the role of *Cdc42bpb* in development across heart, lung, and diaphragm could improve our understanding of key gene pathways, cell–cell communication, and protein dynamics that lead to the co-morbidities found across all three tissues.

Presented are two novel CDH mouse models selected to determine whether the two genes likely contributed to severe, isolated, dorsal CDH in two unrelated patients [5]. Both mouse models present with CDH, though as shown, they differ in both location and phenotypic severity. This key difference appears to be due to differences in underlying cellular mechanisms, as loss of *Cdc42bpb* appears to lead to a delay and failure of the diaphragm muscle to reach the ventral edge of the diaphragm, whereas the *Cnot1^R623W^* allele leads to a subtle change in transcription. Though demonstration of the orthologous patient variant leading to CDH is crucial for validation of human genetic studies, further mechanistic investigation into the role of *Cnot1* in the diaphragm will be difficult. Outstanding questions remain for both genes, including which cell populations are affected, whether gene expression is required throughout diaphragm development, and whether the gene plays similar roles in other organ systems. Similar questions remain for many other CDH candidate genes [1, 5, 6]. Groups have begun to explore diaphragm gene regulation at the single cell level early in development [52] and after diaphragm development is complete [53]. Continued exploration of cell population dynamics across diaphragm development is crucial in understanding the genes presented here and the roles of future validated CDH genes. Combining insights gained from initial validating screens, such as the F0 platform used for the *Cdc42bpb* patient mutation, ‘omics approaches to understand how the gene functions during diaphragm development, and targeted genetic manipulation of the gene all remain critical in understanding both CDH and congenital anomaly pathogenesis and translating these findings into improved clinical diagnostics.

## Materials and methods

### Mice

All animal studies were performed in accordance with the Guide to the Care and Use of Laboratory Animals and were reviewed and approved by the Institutional Animal Care and Use Committee of The Jackson Laboratory.

### Model engineering

The *Cdc42bpb^em1(IMPC)J^* allele was created at The Jackson Laboratory as part of the Knockout Mouse Phenotyping Program (KOMP2) [35]. Guide RNA sequences and allele design details can be found at https://www.mousephenotype.org/data/alleles/MGI:2136459/em1(IMPC)J?alleleSymbol = em1(IMPC)J#mice. Mice were maintained on a C57BL/6NJ (The Jackson Laboratory, strain #005304) background. Mice and embryos were produced through heterozygous by heterozygous matings, and observed vs expected wean genotype numbers were compared using χ^2^ analysis, with a 1:2 expected ratio for wildtype: heterozygous, since homozygous animals were rarely found at wean.

The *Cnot1^em1Murr^* allele was generated using CRISPR/Cas9 endonuclease-mediated genome editing. Guide RNA (CAAGTCCACCCAAAATAGAA) was selected to cleave DNA within exon 16 of the Cnot1 gene and a single stranded oligo donor with a missense R623W (AGG>TGG) and silent C624C (TGC>TGT) variant were introduced to facilitate HDR-mediated genome editing. Potential off-target editing sites were generated using Benchling built-in CRISPR design tools, which utilizes the off-target scores developed by [54], see Supplemental Table 7. The guide and single stranded donor were introduced to single cell C57BL/6NJ zygotes and transferred to pseudo pregnant females. Progeny were screened by DNA sequencing to identify correctly targeted pups, which were then bred to C57BL/6NJ (Stock No. 05304) mice for germline transmission. The C57BL/6NJ-*^Cnot1em1Murr^*/Murr colony was backcrossed to C57BL/6NJ for at least two generations. Screened embryos and neonates were generated across multiple generations, ensuring phenotypes found are linked to the R623W variant continually selected for in matings. See Supplemental Table 8 for oligonucleotide sequences used.

### Embryo and neonate collection

Mouse embryos and neonate pups were collected from heterozygous-by-heterozygous timed matings for both *Cdc42bpb* and *Cnot1* lines. DNA was extracted from either yolk sacs (embryos) or tail tips (neonate pups) for genotyping by melt curve analysis for *Cnot1^R623W^* or standard PCR for *Cdc42bpb^−^* (see primers in supplemental table 7). Embryos were harvested through microdissection, imaged for gross morphological phenotypes on a M125 Leica dissecting microscope, crown-rump length (CRL) measurements for stage confirmation were taken, notable external phenotypes were noted and embryos/neonates were either: exsanguinated in 1× PBS with 0.2 mg/mL heparin (for E16.5-18.5) then fixed overnight in 4% paraformaldehyde (PFA) at 4°C; directly fixed overnight if younger; or neonates were euthanized by decapitation, then fixed in 4% PFA. To score herniations diaphragms were micro-dissected after fixation as previously described [8], for *Cnot1* diaphragms liver was also removed to more easily visualize diaphragm abnormalities. To quantify *Cnot1* herniation size the total area of the herniation was divided by the total area of the diaphragm muscle from M1654 Leica dissecting microscope images. Herniation percentages between *Cnot1^+/R623W^* and *Cnot1^R623W/R623W^* were statistically compared using Student’s T test with GraphPad Prism version 10.0.0 for Windows, GraphPad Software, Boston, Massachusetts USA, www.graphpad.com. Post-hoc power analysis for herniation penetrance in *Cnot1^+/R623W^* and *Cnot1^R623W/R623W^* embryos was calculated using the pwr R package (version 1.3-0).

### *Cdc42bpb* ^*−/−*^ neonate survival screen

Expected birth date was determined based on seminal plug in *Cdc42bpb^−/+^* timed matings, and boxes were checked every two hours throughout the day. Once pups were born and discovered, CRL and body weight were measured and toe clips were taken to both identify each pup and genotype each pup using PCR amplification. Litters were checked every two hours after initial discovery and measurements were repeated every four hours.

### μCT embryo preparation, scanning and analysis

After 4% PFA overnight fixation samples were washed in PBS and placed in STABILITY buffer [35] for 5–7 days at 4°C. Embryos were then incubated at 37°C for 4 hours to polymerize the STABILITY buffer, which was then cleaned off the samples. Embryos were then placed in 50 mL iodine-based Lugol’s Solution and incubated with agitation at room temperature for 5–7 days. Counterstained samples were then mounted in 1% agarose in 5 mL flat bottom conical tubes, stacking two samples vertically. Mounted, counterstained samples were imaged on either a Bruker Skyscan 1172 or 1272 μCT (Bruker Biospin), with a 0.5 millimeter filter, 2048x2048 resolution, and pixel size of 11 μm (parameters adapted from [35]). Scan image sets were then reconstructed using NRecon software (Bruker), exporting data as transaxial bmp image stacks, which were then converted into.nrrd three-dimensional image files using Fiji (ImageJ). ITK-SNAP [36] software was used to open.nrrd files, score internal structural tissue defects and render diaphragm and lungs for further phenotyping. Lung relative volumes were collected by dividing rendered lung voxel volumes from total rendered embryo/neonate voxel volumes in ITK-SNAP. For *Cdc42bpb* rendered diaphragms, ventral gap distance measurements were taken by exporting diaphragm 3D renders to 3DSlicer and using the measure tool across the two edges of muscle at the midpoint between the ventral body wall and the central tendon (see Supplemental Fig. 1). Heart defects were scored by comparing coronal and transverse sections of *Cdc42bpb^−/+^* and *Cdc42bpb^−/−^* μCT scanned hearts to *Cdc42bpb^+/+^* scanned litter mates. Kaplan–Meier survival plot and analysis were performed using GraphPad Prism version 10.0.0 for Windows, GraphPad Software, Boston, Massachusetts USA, www.graphpad.com.

### Patient variant modeling using F0 embryo CRISPR/Cas9 editing

CRISPR gRNA targeting the mouse orthologous *Cdc42bpb* CDH patient variant location and single strand DNA carrying both patient missense variant and gRNA-disrupting base pair change were designed using design tools built-in to the Benchling platform (benchling.com, [55, 56]). Potential off-target editing sites were generated using Benchling built-in CRISPR design tools, which utilizes the off-target scores developed by [55], see Supplemental Table 7. CRISPR/Cas9 ribonucleotide complexing, E0.5 electroporation of editing components and implantation were carried out using previously described methods [57]. CRISPR/Cas9 components were ordered from Integrated DNA Technologies (IDT) from the Alt-R Cas9 platform. E18.5 embryos were harvested from pseudopregnant females implanted with edited fertilized zygotes, yolk sacs were collected for DNA, and embryos were fixed and processed for μCT scanning using the same methods as embryos collected from timed matings. Embryos were scanned, scored for structural defects and diaphragms were rendered with ITK-SNAP [36]. Diaphragm ventral gaps were measured using the same methods in 3DSlicer as timed matings. PCR amplified DNA was Sanger sequenced and trace files were deconvoluted using ICE (ICE CRISPR Analysis. 2025. v3.0. EditCo Bio) and percentages of variant knock-in and knockout were determined.

### Western blotting

E15.5 diaphragms were microdissected from harvested *Cdc42bpb^−/+^* x *Cdc42bpb^−/+^* as previously described [58], snap frozen in liquid nitrogen and stored at −80°C. Once embryos were genotyped through standard PCR, like genotypes were pooled to three diaphragms per tube (e.g. per sample [N] in final quantification), then lysed using RIPA buffer (Thermo Fisher Scientific) with cOmplete™ Protease Inhibitor (Sigma-Aldrich) and mechanically disrupted with a disposable plastic pestle (Thermo Fisher Scientific). Protein supernatant was separated by centrifugation at 14 000 rpm for 20 minutes at 4°C. Protein concentration was determined with Pierce™ BCA protein assay kit (Thermo Fisher Scientific). Protein was diluted to a concentration of 1 μg/μL in RIPA and 4× Laemmli Sample Buffer (Bio-Rad) and 30 μL per sample was loaded into Mini-PROTEAN® TGX™ Precast Protein Gels, 4–20% (Bio-Rad). Protein was run through the gel at a constant 105 volts, with Precision Plus Protein™ Dual Color Standards, 10–250 kDa (Bio-Rad) included in the first well. Protein was then transferred to a PVDF membrane at 4°C running a constant 100 volts for 1 hour. The PVDF membrane was blocked for 1 hour in TBST (tris-buffered saline with 0.05% Tween) with 3% BSA (bovine serum albumin, Sigma-Aldrich) and then incubated overnight in block solution with rabbit anti-CDC42BPB (1:200 Novus Biologicals, NBP1-81440) and mouse anti-beta ACTIN (1:5000 Invitrogen, MA5-15739) at 4°C with agitation. The membrane was then washed with TBST, incubated with HRP-conjugated secondaries, goat anti-rabbit (1:1000 Bio-Techne, HAF008) and goat anti-mouse (1:5000 Invitrogen, G21040), for 1 hour at room temperature and then washed again with TBST. Bands were then visualized using Pierce™ SuperSignal™ West Pico PLUS Chemiluminescent substrate (Thermo Scientific) and imaged with a G:Box Chemi XRQ blot imager (Syngene). Band intensities were then quantified in Fiji (ImageJ) by measuring greyscale intensity, subtracting background (unstained membrane) and normalizing CDC42BPB band intensity to control β-ACTIN band intensity. Supplemental fig. 9 shows an example uncropped blot, with bands labeled. Quantifications of bands near CDC42BPB also impacted by genotype included, showing significant decrease in *Cdc42bpb^−/−^* samples. Genotype normalized band intensities were then statistically compared using an unpaired Student’s *t-*test using GraphPad Prism version 10.0.0 for Windows, GraphPad Software, Boston, Massachusetts USA, www.graphpad.com.

### Colorimetric myosin staining

E13.5-E16.5 embryos were collected from *Cdc42bpb^−/+^* timed matings, fixed in 4% PFA overnight at 4°C, then micro-dissected to expose the cranial side of the diaphragm, as previously described [8]. Embryos were then bleached for 2 hours at room temperature in Dent’s bleach (54% methanol, 33% H_2_O_2_, 13% DMSO), washed with 100% methanol and stored in Dent’s fix (80% methanol, 20% DMSO) for a minimum of two weeks at 4°C. After extended fixation embryos were washed with PBS, including a one-hour wash at room temperature, then endogenous alkaline phosphatases were inactivated by incubating in PBS at 65°C. Embryos were then washed in room temperature PBS, blocked for one hour in 5% goat serum (ThermoFisher Scientific), 20% DMSO and 75% PBS, then transferred to fresh block with Monoclonal Anti-Myosin (skeletal, fast)-Alkaline Phosphatase Sigma #A4335 Mouse IgG1 at a dilution of 1:150 and incubated at room temperature for two days. Embryos were then washed with PBS, then in NTMT (100 mM NaCl, 100 mM Tris–HCl pH 9.5, 50 mM MgCl_2_). Embryos were then stained with a 50% NTMT, 50% 1-Step™ NBT/BCIP substrate solution (Thermo Scientific) for 30 minutes, then post-fixed in 4% PFA/0.1% glutaraldehyde. Stained diaphragms were then imaged on Leica M125 dissecting microscopes and ventral gaps were measured in Fiji (ImageJ), drawing lines between muscle boundaries at the midpoint between the central tendon and mid-ventral body wall. Gap distances were statistically compared across genotypes at each timepoint using one-way ANOVA with multiple comparisons using GraphPad Prism version 10.0.0 for Windows, GraphPad Software, Boston, Massachusetts USA, www.graphpad.com.

### Developing lung immunofluorescent staining

E13.5 embryos were collected from *Cdc42bpb^−/+^* timed matings, lungs were micro-dissected and fixed for 15 minutes in 4% PFA on ice. Fixed lungs were then washed with PBS and PBS with 0.1% TritonX-100 (PBSTx) (Thermo Scientific), blocked in PBSTx with 10% heat-inactivated sheep serum and incubated with Monoclonal Anti-Cytokeratin, pan antibody produced in mouse (1:250 Sigma-Aldrich, C2931) in block solution overnight at 4°C. Lungs were then washed in PBSTx at 4°C and incubated with anti-MsIgG1 dylight488 secondary antibody at a dilution of 1:250 in block solution in 4°C overnight. Lungs were then washed in PBSTx at 4°C, then imaged on a Leica M165FC fluorescent dissecting microscope. Distal buds were then counted using Fiji (ImageJ), then genotypes were compared statistically using one-way ANOVA with multiple comparisons using GraphPad Prism version 10.0.0 for Windows, GraphPad Software, Boston, Massachusetts USA, www.graphpad.com.

### Bulk RNA sequencing and analysis

Embryos were collected from *Cnot1^+/R623W^* x *Cnot1^+/R623W^* timed matings and diaphragms were micro-dissected as previously described [58], snap frozen in liquid nitrogen and stored at −80°C. Total RNA was isolated from tissue using the Qiagen miRNeasy micro RNA Isolation Kit (Qiagen). Tissues were homogenized in TRIzol (ThermoFisher) using a Pellet Pestle Motor (Kimbal). After the addition of chloroform, the RNA-containing aqueous layer was removed for RNA isolation according to the manufacturer’s protocol. RNA concentration and quality were assessed using the Nanodrop 2000 spectrophotometer (Thermo Scientific) and the RNA 6000 Nano Assay (Agilent Technologies). Stranded libraries were constructed using the KAPA RNA Hyper Prep Kit with RiboErase (HMR) (Roche Sequencing and Life Science), according to the manufacturer’s protocol. Briefly, the protocol entails depletion of ribosomal RNA (rRNA), RNA fragmentation, first and second strand cDNA synthesis, ligation of Illumina-specific adapters containing a unique barcode sequence for each library, magnetic bead size selection, and PCR amplification. The quality and concentration of the libraries were assessed using the D5000 ScreenTape (Agilent Technologies) and Qubit dsDNA HS Assay (ThermoFisher), respectively, according to the manufacturers’ instructions. Libraries were sequenced 150 bp paired-end on an Illumina NovaSeq 6000 using the S4 Reagent Kit v1.5. RNA sequencing reads were batch processed using The Jackson Laboratory standard Nextflow RNAseq pipeline (https://github.com/TheJacksonLaboratory/jds-nf-workflows/wiki/RNA-Pipeline-ReadMe), aligning to the mm10 (GRCm38) mouse genome and GENCODE gene annotation release M25 (GRCm38.6). In brief, sequenced reads were trimmed with Trim Galore (https://github.com/FelixKrueger/TrimGalore), aligned with STAR [59] and counts were quantified with Salmon [60] and StringTie [61]. Differential gene and transcript analysis was performed with DESeq2 [62], using standard parameters. Statistically significant differentially expressed genes and transcripts per comparison were analyzed with STRING DB (https://string-db.org/) with standard parameters. Alternative polyadenylation between genotypes was determined using APALYZER 3’ UTR analysis standard parameters [42]. Volcano plots comparing RED to -Log_10_(P-value) were generated by APALYZER. RED to -Log_10_(Adjusted P-value) were generated using ggplot2 [63]. RNA sequencing data is available on GEO at accession number GSE318891.

## Supplementary Material

Supplementary_Data_ddag106
