# Supplementary material for: Modeling patient variants of Cnot1 and Cdc42bpb results in distinct forms of congenital diaphragmatic hernia in mice
Source: Hum Mol Genet. 2026 Oct 14;35(21):ddag106. doi: 10.1093/hmg/ddag106 (PMC13665469; doi:10.1093/hmg/ddag106)
Supplement: Supplementary file 1 [file supplementary_data_ddag106.zip › 092926Supplemental_materialrevised.pdf]

Supplementary information

This file includes:

Supplementary Figures 1-9

Descriptions of Supplementary Tables 1-8

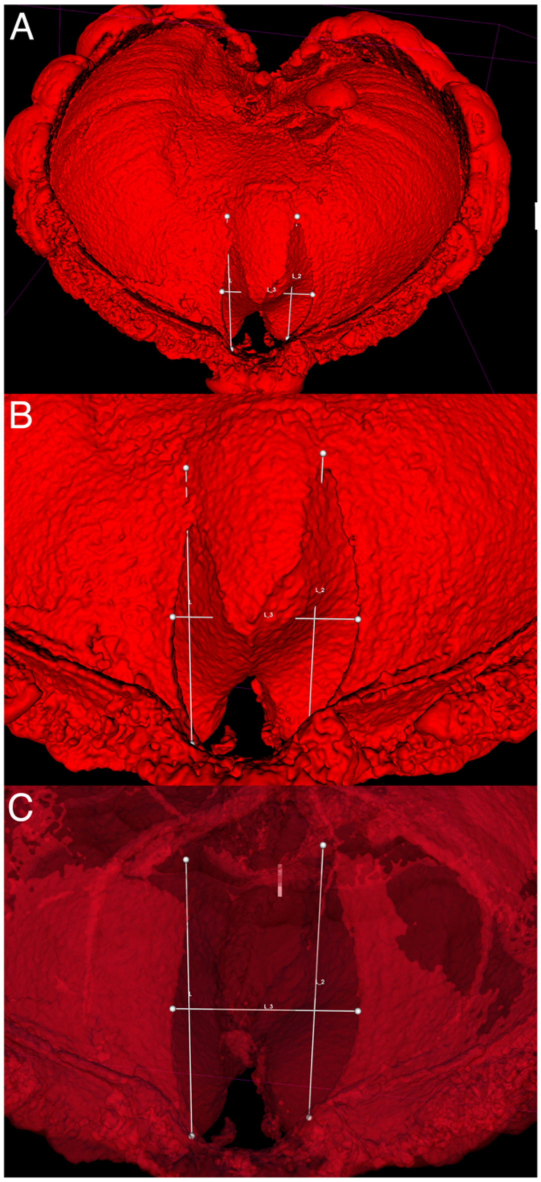

Supplemental Figure 1: Placement of 3Dslicer markers used for measuring diaphragm ventral gaps. Markers are placed at the medial edge of the migrated diaphragm muscle and connected to the distal body ventral body wall (white dots and vertical lines). The midpoint of the left dorsal-ventral line is found, with a landmark node placed on the edge of the diaphragm muscle. A line from this node to the opposing edge of the diaphragm muscle is created, staying perpendicular to the vertical lines. The bisection with the right vertical line is then confirmed to be within ±20% of the midpoint of the right vertical line. The length of this line is then taken as the gap distance. A) Full diaphragm render view. B) Zoom into ventral gap. C) Render volume opacity at 50% for easier viewing of landmarks and lines.

Alt text: 3D renderings of micro-CT diaphragm with landmark points and lines demonstrating how ventral gaps were measured.

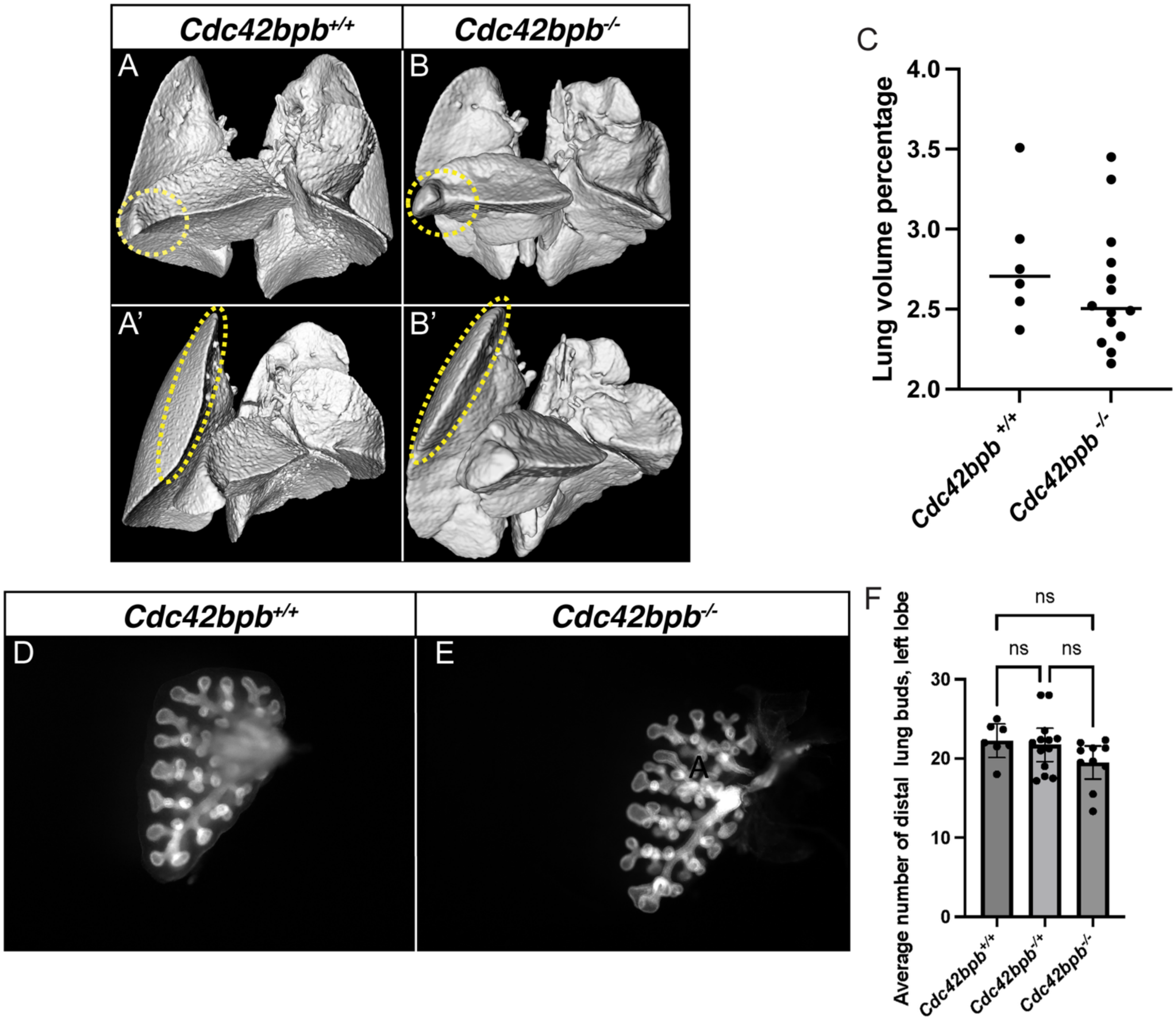

Supplemental Figure 2: Lung morphology of *Cdc42bpb*$^{-/-}$ mutants. Representative μCT surface renderings of E18.5 lungs A) *Cdc42bpb*$^{+/+}$ and B) *Cdc42bpb*$^{-/-}$ mutants, showing dysmorphic features including blunted distal edges of lung lobes and accessory lobe horn (yellow dashed-lines). C) Comparison of lung volume percentage (rendered lung volume/whole embryo render volume) between *Cdc42bpb*$^{+/+}$ and *Cdc42bpb*$^{-/-}$, which were not significant (Student's t-test). D, E) Whole mount pan-cytokeratin immunofluorescence of E13.5 left lung lobes, show similar lung branching pattern between *Cdc42bpb*$^{+/+}$ controls and *Cdc42bpb*$^{-/-}$ mutants. F) Quantitation of number of distal buds for each genotype (not significant by one-way ANOVA). Error bars represent 95% Confidence Intervals.

Alt text: 3D rendered lungs from Cdc42bpb null embryos showing differences in morphology accompanied by plots showing lung volume and distal lung bud number, counted from representative immunofluorescent images shown, do not change.

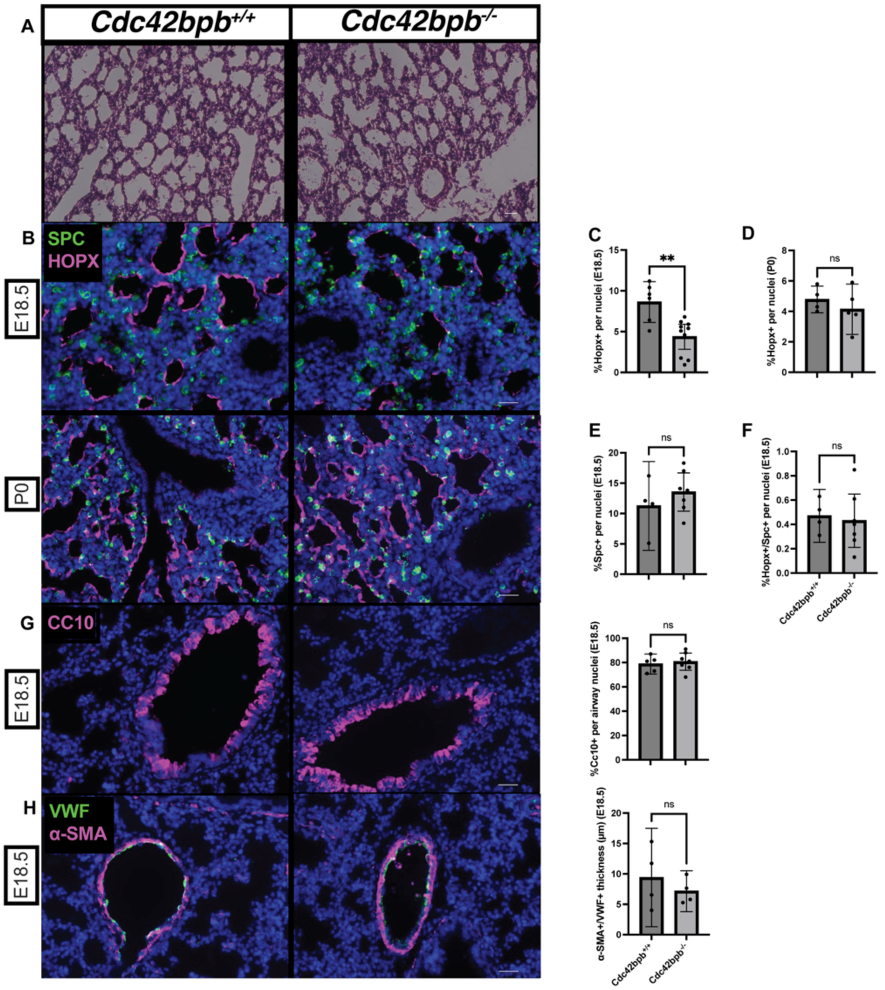

Supplemental Figure 3: E18.5 *Cdc42bpb*$^{-/-}$ mice exhibit a delay in ATI cell differentiation. A) H&E stainings of E18.5 *Cdc42bpb*$^{+/+}$ and *Cdc42bpb*$^{-/-}$ mice. B) Immunostainings of alveolar subpopulations in *Cdc42bpb*$^{+/+}$ and *Cdc42bpb*$^{-/-}$ mice at E18.5 and P0. C) Quantification of E18.5 HOPX+ ATI cell population reduction in *Cdc42bpb*$^{+/+}$ and *Cdc42bpb*$^{-/-}$ mice (p= 0.0051, T-test). D) Quantification of P0 HOPX+ ATI population in *Cdc42bpb*$^{+/+}$ and *Cdc42bpb*$^{-/-}$ mice (p=0.3784). E) Quantification of E18.5 SPC staining of the ATII population in *Cdc42bpb*$^{+/+}$ and *Cdc42bpb*$^{-/-}$ mice (p=0.4238, T-test). F) Quantification of E18.5 double-positive ATI/ATII transitional cell population in *Cdc42bpb*$^{+/+}$ and *Cdc42bpb*$^{-/-}$ mice (p=0.7310). G) Immunostainings and quantification of CC10+ club cell populations of E18.5 *Cdc42bpb*$^{+/+}$ and *Cdc42bpb*$^{-/-}$ mice (p=0.6557). H) Vascular smooth muscle and endothelial cell immunostainings with quantification of blood vessel thickness in E18.5 *Cdc42bpb*$^{+/+}$ and *Cdc42bpb*$^{-/-}$ mice (p=0.4617). **=p<0.01 by Student's t-test. Error bars represent 95% Confidence Intervals. Scale bar – 25μm

Alt text: Representative immunofluorescent and histology stains of Cdc42bpb lungs showing expression of cell type markers, with quantification plots included for each marker.

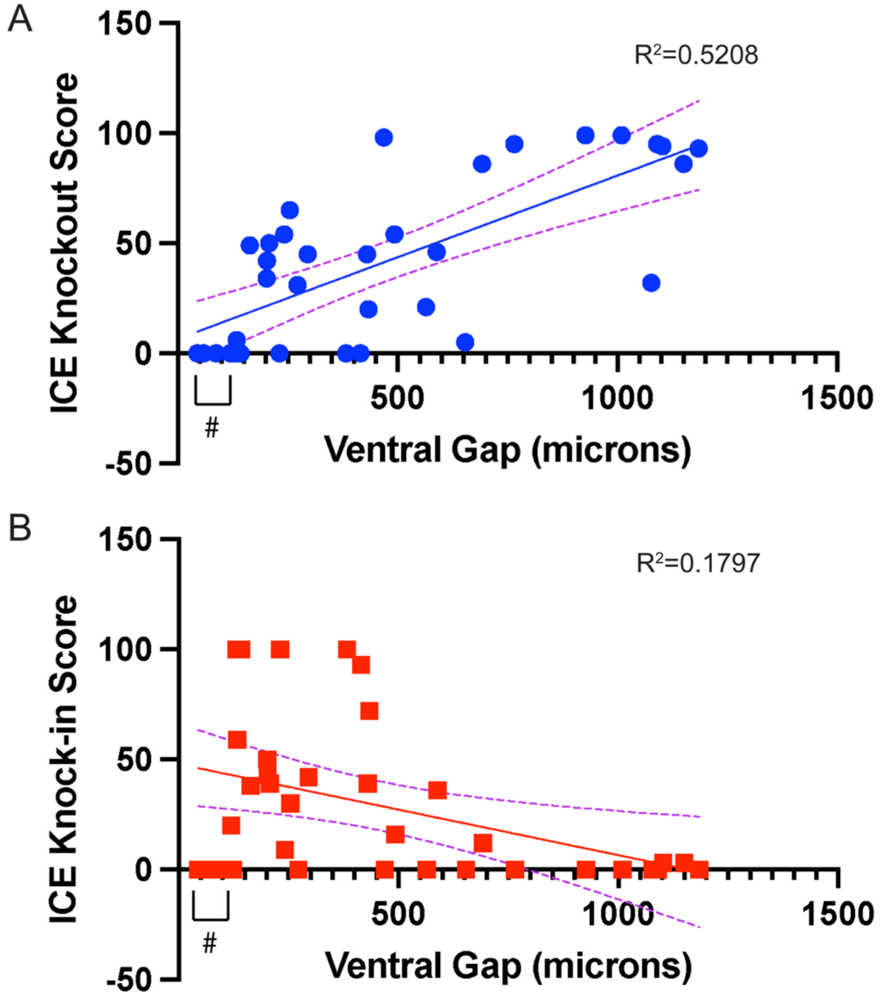

Supplemental Figure 4: Correlations of ventral diaphragm gap distances with amount of *Cdc42bpb* knockout (A) and *Cdc42bpb*^{T1179M} knock-in (B). R-squared values are listed for each linear regression (solid lines), along with 95% confidence interval (dashed lines). # denotes unedited embryos on each plot.

Alt text: Scatterplots showing correlation of Cdc42bpb ventral gap to ICE scores in F0 experiment embryos.

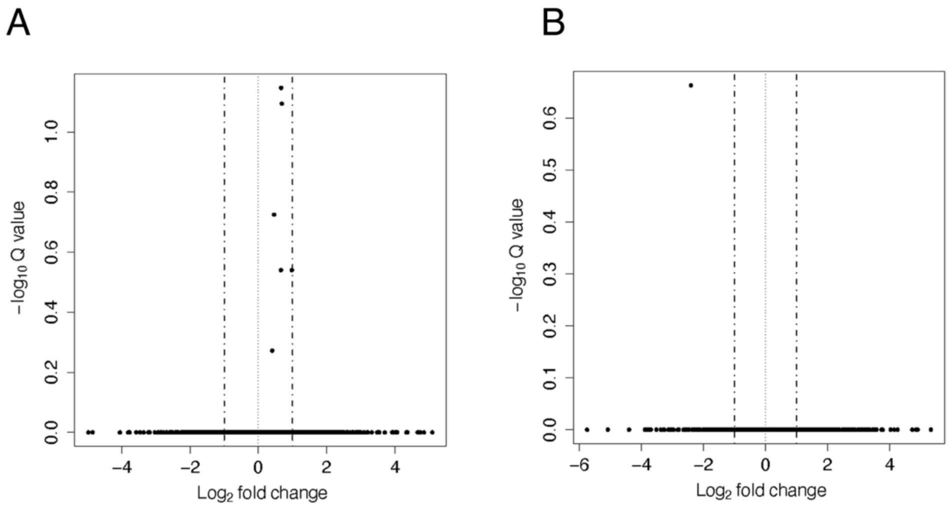

Supplemental Figure 5: No differentially expressed genes are found between *Cnot1* mutant embryos and *Cnot1*$^{+/+}$controls at E13.5. Volcano plots of differentially expressed genes between *Cnot1*$^{+/+}$ and A) *Cnot1*$^{+/R623W}$ or B) *Cnot1*$^{R623W/R623W}$ diaphragms. No genes pass p-adjusted (Q) value threshold of 0.05 in either comparison.

Alt text: Volcano plots of E13.5 gene differential expression, showing no genes are significant in either genotype comparison

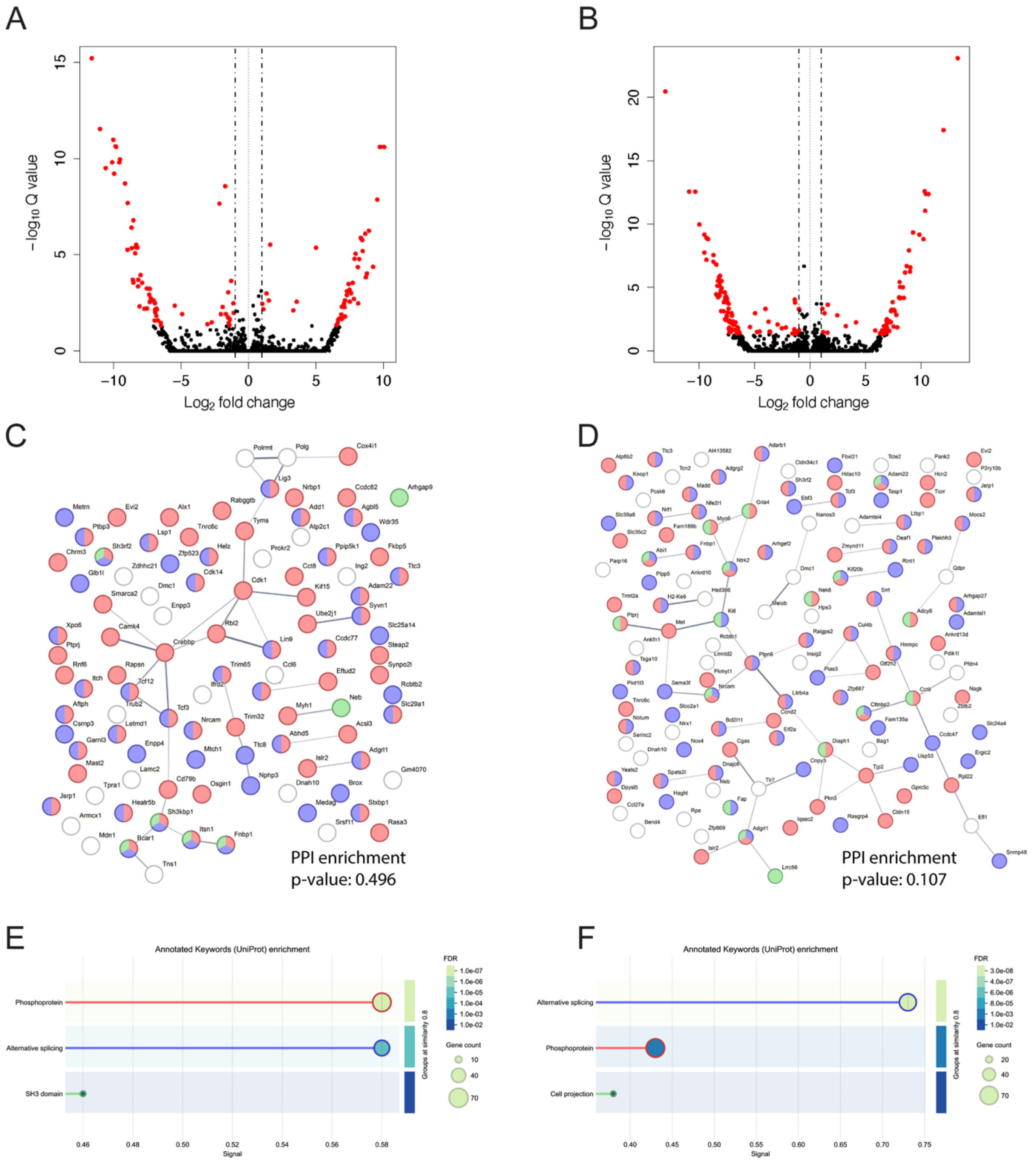

Supplemental Figure 6: Transcript differential expression analysis of E13.5 diaphragms. Volcano plots of transcript differential analysis between A) *Cnot1*$^{+/+}$ vs. *Cnot1*$^{+/R623W}$ and B) *Cnot1*$^{+/+}$ vs. *Cnot1*$^{R623W/R623W}$. Transcripts (datapoints) with absolute $\log_2$ fold change greater than 1 and p-adjusted (Q) values less than 0.05 highlighted in red. STRING gene networks of significant differentially expressed transcripts from C) *Cnot1*$^{+/+}$ vs. *Cnot1*$^{+/R623W}$ and D) *Cnot1*$^{+/+}$ vs. *Cnot1*$^{R623W/R623W}$ comparisons. Lines between nodes denote interaction between genes with STRING interaction confidence scores greater than or equal to 0.4. E) and F) show Uniprot terms enriched in transcript networks for C) and D), respectively, by STRING GO analysis. Genes belonging to each Uniprot term are shown fully or partially colored in C) and D) as terms in E) and F).

Alt text: Volcano plot, gene interaction network and gene ontology results for E13.5 *Cnot1* genotype comparisons of transcript isoform differential expression.

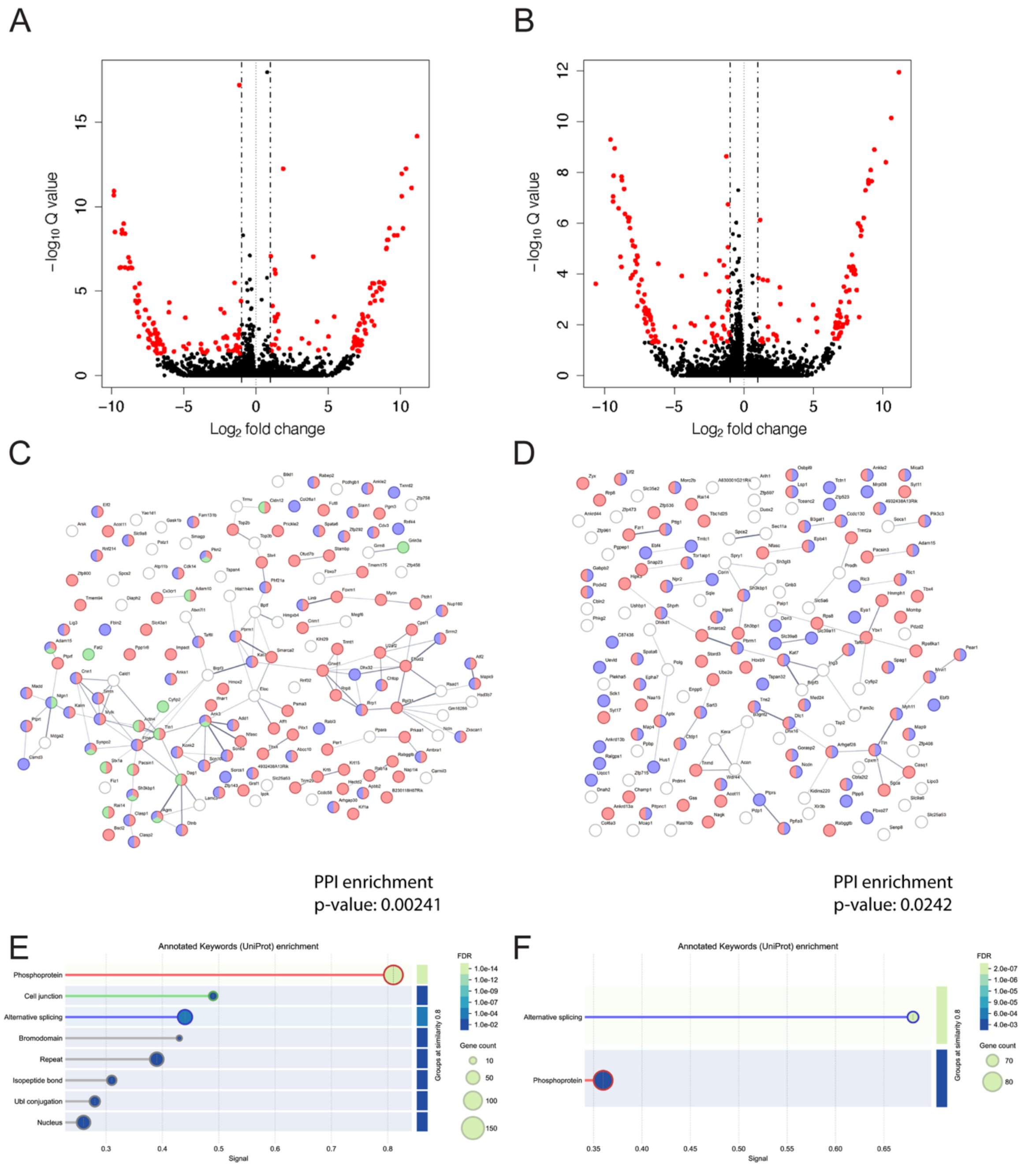

Supplemental Figure 7: Transcript differential expression analysis of E14.5 diaphragms. Volcano plots of transcript differential analysis between A) *Cnot1*$^{+/+}$ vs. *Cnot1*$^{+/R623W}$ and B) *Cnot1*$^{+/+}$ vs. *Cnot1*$^{R623W/R623W}$. Transcripts (datapoints) with absolute $\log_2$ fold change greater than 1 and p-adjusted (Q) values less than 0.05 highlighted in red. STRING gene networks of significant differentially expressed transcripts from C) *Cnot1*$^{+/+}$ vs. *Cnot1*$^{+/R623W}$ and D) *Cnot1*$^{+/+}$ vs. *Cnot1*$^{R623W/R623W}$ comparisons. Lines between nodes denote interaction between genes with STRING interaction confidence scores greater than or equal to 0.4. E) and F) show Uniprot terms enriched in transcript networks for C) and D), respectively, by STRING GO analysis. Genes belonging to each Uniprot term are shown fully or partially colored in C) and D) as terms in E) and F).

Alt text: Volcano plot, gene interaction network and gene ontology results for E14.5 Cnot1 genotype comparisons of transcript isoform differential expression.

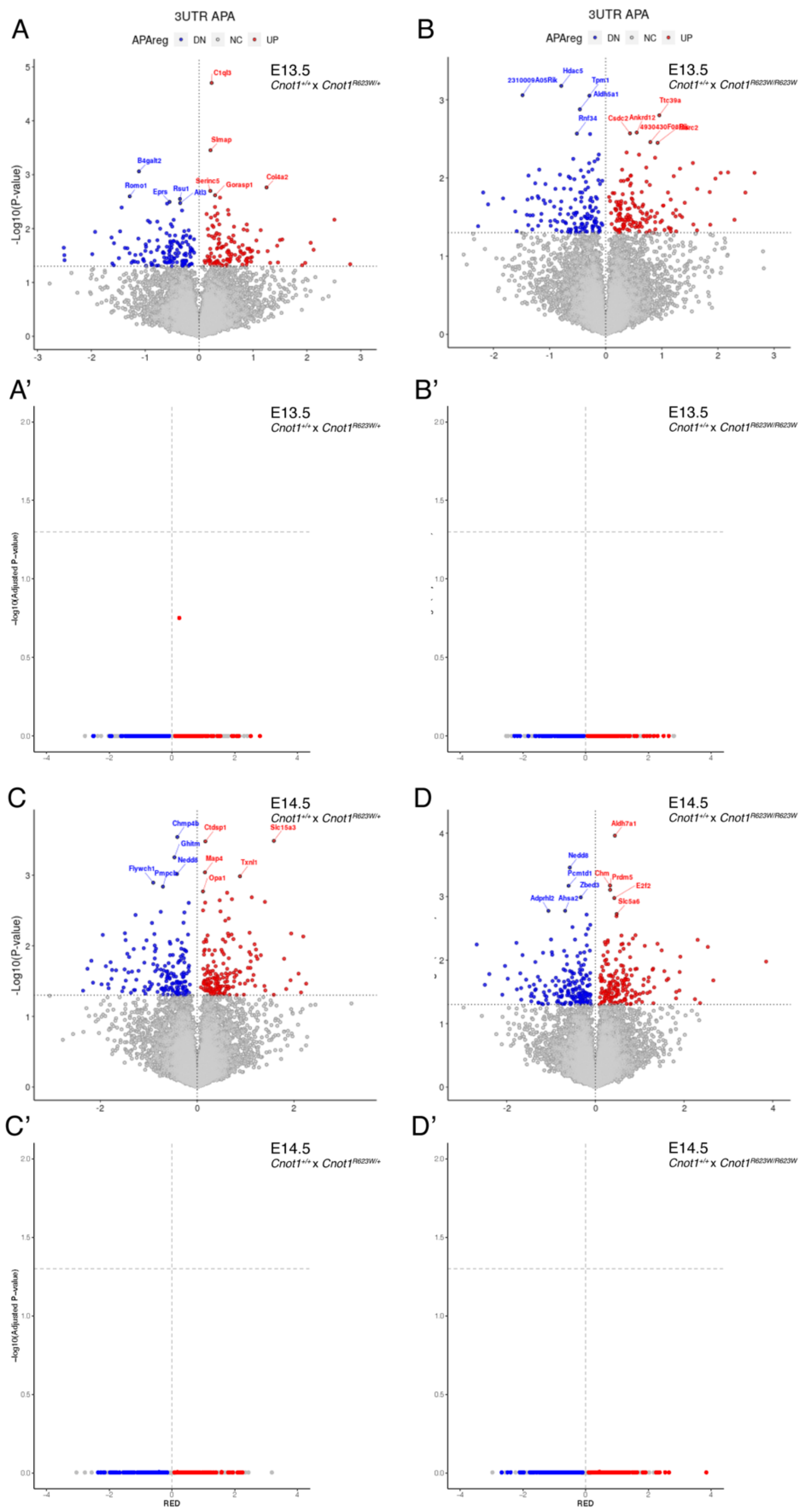

Supplemental Figure 8: Alternative polyadenylation analysis. Genes with differential polyadenylation comparing mutant *Cnot1* samples against wildtype at E13.5 (A-B) and E14.5 (C-D). Apalyzer generated APA relative expression score differences (RED) between genotypes compared are plotted on the X-axis (A-D). Normalized -$\log_{10}$ of either Apalyzer generated P-value (A, B, C ,D) or multiple test corrected p-adjusted value (A', B', C', D'), as determined by Student's T-test with Bonferroni correction, plotted on Y-axis. Red datapoints represent genes with increased 3' UTR polyadenylation (UP), blue datapoints with decreased 3' UTR polyadenylation (DN) and grey datapoints represent non-significantly changed based on p-value <0.05 (NC). Top ten differential genes labeled in A, B, C, and D.

Alt text: Volcano plots of transcripts with alternative polyadenylation comparing wildtype to one or two copies of the *Cnot1* variant at both embryonic stages. When Adjusted P-Value is plotted no transcripts are significant.

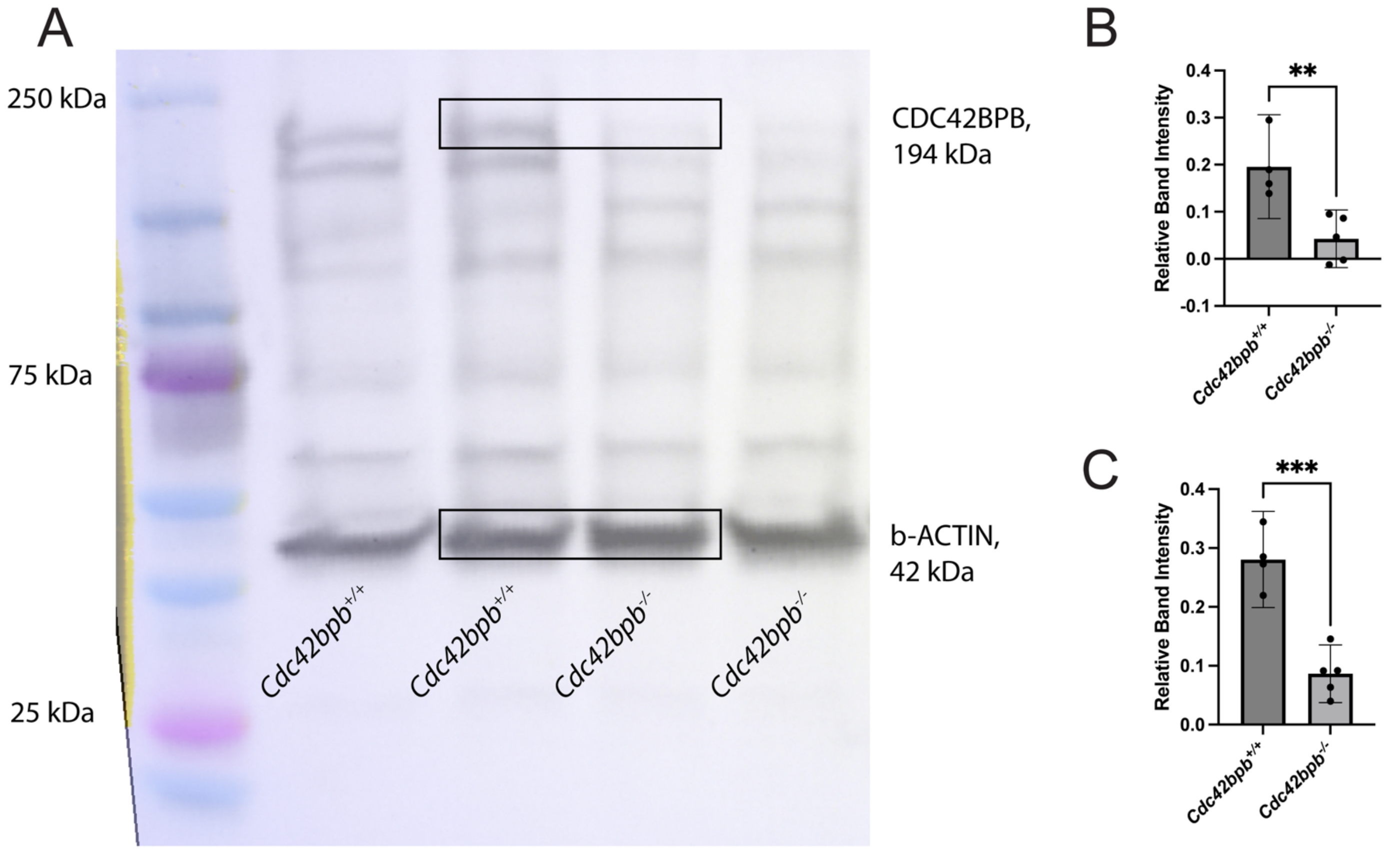

Supplemental Figure 9: Full Western blot comparing CDC42BPB expression in *Cdc42bpb*$^{+/+}$ and *Cdc42bpb*$^{-/-}$ E15.5 diaphragms. A) Full blot image with lanes labeled per genotype, b-ACTIN loading control and CDC42BPB bands labeled. Highlight boxes show bands used in Figure 1D. Bio-Rad Precision Plus Protein Dual Color ladder used in lane 1. Quantification of bands below the 194 kDa CDC42BPB band (B) and combining both bands (C), which are also impacted by genotype. Both intensity band quantifications normalized to beta-ACTIN. ** p-value <0.01, *** p-value <0.001 by Student's t-test, 95% CI error bars shown.

Alt text: Western blot of two WT and two Cdc42bpb knockout diaphragm samples stained with anti-Cdc42bpb and beta-actin antibodies. Boxes highlight Cdc42bpb bands at 194 kDa and beta-actin at 42 kDa shown in Figure 1. Panels B and C show bar plots of the band intensity of the lower Cdc42bpb band and the combination of bands for WT and mutant samples.

Supplemental Table 1: ICE generated CRISPR editing scores for C>T p.T1179M *Cdc42bpb* F0 embryos.

Supplemental Table 2: DESeq2 results of gene level comparisons between RNA-sequencing samples.

Supplemental Table 3: STRING DB significant ontology terms for each set of significantly differentially expressed genes across sample comparisons.

Supplemental Table 4: DESeq2 results of transcript level comparisons between RNA-sequencing samples.

Supplemental Table 5: STRING DB significant ontology terms for each set of significantly differentially expressed transcripts across sample comparisons.

Supplemental Table 6: Apalzyer results comparing $Cnot1^{+/+}$ to either $Cnot1^{+/R623W}$ or $Cnot^{R623W/R623W}$ at either E13.5 or E14.5.

Supplemental Table 7: CRISPR guide off-target analysis for single guide edits targeting $Cnot1^{R623W}$ and $Cdc42bpb^{T1179M}$.

Supplemental Table 8: List of oligonucleotide sequences used in this study.
